# Intravital molecular imaging reveals that ROS-caspase-3-GSDME-induced cell punching enhances humoral immunotherapy targeting intracellular tumor antigens

**DOI:** 10.7150/thno.75966

**Published:** 2022-10-31

**Authors:** Bolei Dai, Ren Zhang, Shuhong Qi, Lei Liu, Xian Zhang, Deqiang Deng, Jie Zhang, Yilun Xu, Fanxuan Liu, Zheng Liu, Qingming Luo, Zhihong Zhang

**Affiliations:** 1 Britton Chance Center and MoE Key Laboratory for Biomedical Photonics, Wuhan National Laboratory for Optoelectronics-Huazhong University of Science and Technology, Wuhan, Hubei 430074, China.; 2 School of Biomedical Engineering, Hainan University, Haikou, Hainan 570228, China.

**Keywords:** intravital molecular imaging, intracellular antigens, humoral immunity, immune complexes, tumor liver metastasis

## Abstract

Tumor antigens (TAs)-induced humoral immune responses or TAs-specific antibodies have great application prospects for tumor therapy. However, more than half of TAs are intracellular antigens (intra-Ags) that are hardly recognized by antibodies. It is worthy to develop immunotherapeutic strategies for targeting intra-Ags.

**Methods:** We used the far-red fluorescent protein tfRFP as an intracellular antigen to immunize mice and generated a liver metastasis model by injecting tfRFP-expressing B16 melanoma cells (tfRFP-B16) *via* the spleen. Intravital molecular imaging and atomic force microscopy were performed to visualize the formation of tfRFP antigen-antibody complexes (also known as immune complexes) and punched holes in cell membranes.

**Results:** The results showed that the tfRFP-elicited immune responses inhibited the metastasis of tfRFP-expressing melanoma cells in the liver. In the circulating tfRFP-B16 tumor cells, elevated reactive oxygen species (ROS) induced slight caspase-3 activation, a probable key factor in the cleavage of gasdermin E (GSDME) proteins and punching of holes in the tumor cell membrane. Increased tumor cell membrane permeability led to the release of intra-Ag tfRFP and binding with anti-tfRFP antibodies. The formation of tfRFP antigen-antibody complexes on the membranes of tfRFP-B16 cells activated complement components to form membrane attack complexes to further destroy the cell membrane. Neutrophils were rapidly recruited, and F4/80^+^ macrophages phagocytized the dying tumor cells.

**Conclusion:** The process of circulating tumor cell elimination in the tfRFP-immunized mice was triggered through the ROS-caspase-3-GSDME pathway to form intra-Ag-antibody immune complexes, which were involved in the activation of the complement system, as well as the recruitment of neutrophils and F4/80^+^ macrophages. An intra-Ag-elicited humoral immune response is a potent strategy for eliminating liver metastasis, which is unaffected by the liver immune tolerogenic status.

## Introduction

Tumor vaccines have been widely reported as a promising therapeutic strategy for inducing a specific anti-tumor immune response 1. Initially, tumor vaccines were designed to induce cellular immune responses against tumors, and tumor antigen (TA) recognized by T lymphocytes was considered as the core of tumor vaccine efficacy 2. TA-specific monoclonal antibodies (mAbs) have been promising for cancer immunotherapy for a long time 3, 4. Therefore, if tumor vaccines can induce a potent humoral immune response, they would also have a huge potential for cancer immunotherapy. At present, an important developmental direction of tumor vaccines is the induction of both humoral and cellular immune responses 1, 5.

The therapeutic effect of TA-specific mAbs or TA-induced humoral immune responses is mainly achieved through antibody-dependent cell-mediated cytotoxicity (ADCC) 6, 7, antibody-dependent cell phagocytosis (ADCP) 8, and complement-dependent cytotoxicity (CDC) 9, 10. TAs are categorized as cell extracellular antigens and intracellular antigens (intra-Ags) based on the site of their localization 11, 12. Most mAbs approved by the Food and Drug Administration (FDA) for cancer immunotherapy until now only target extracellular TAs 13, even if more than half of TAs are intracellular antigens 12. The mAbs that target intra-Ags are difficult to use in cancer immunotherapy 7, 14, primarily because mAbs cannot penetrate the cell membrane to recognize intra-Ags 15. Therefore, immunotherapeutic strategies or tumor vaccines need to be developed by targeting intra-Ags, which will broaden the application of humoral immunotherapy.

For humoral immunotherapy against intra-Ag-expressed tumor cells, the first step is to facilitate antibody recognition of intra-Ags. Breaching the integrity of cell membranes could be an effective way to achieve this goal. The cell membrane can be destroyed in several ways, including through punching holes in cell membranes (by gasdermin family proteins 16, 17, complement membrane attack complexes (MACs) 18-20, perforin 21, etc.), membrane lipid peroxidation-related ferroptosis, cell necrosis, and so on 22, 23. Consequently, we speculated that if some factors can increase the permeability of the tumor cell membrane at the early stage of tumor development or metastasis, an intra-Ag-elicited humoral immune response or intra-Ag-specific mAbs will ensure anti-tumor therapeutic efficiency.

Apparently, reactive oxygen species (ROS) produced by circulating tumor cells increase during tumor cell metastasis, inducing tumor cell apoptosis 24-26. In the presence of GSDME, caspase-3 activation in cells results in the cleavage of GSDME proteins to GSDME-N, which can punch holes in cell membranes 16. Hence, it is worth knowing whether the rise in ROS levels of tumor cells during metastasis provides opportunities for the binding of antibodies to intra-Ags before initiating tumor clearance in intra-Ag-elicited humoral immunity.

The liver has a high incidence of tumor metastasis due to its immune tolerogenic microenvironment 27. We previously developed an effective fluorescence model antigen system based on the tetrameric far-red fluorescent protein KatushkaS158A (tfRFP), which elicits both humoral and cellular immunity in C57BL/6 mice 28. The growth of tfRFP-expressing melanoma B16 (tfRFP-B16) cells with subcutaneous *in situ* inoculation was efficiently suppressed in tfRFP-immunized C57BL/6 mice. During tumor elimination, a large number of tfRFP^+^ microparticles were generated in the tumor microenvironment, forming antigen-antibody complexes due to the tfRFP-elicited humoral immune response 28. Thus, tfRFP-immunized mice inoculated with tfRFP-B16 cells provided a suitable model for investigating the antitumor ability of an intra-Ag-elicited humoral immune response. Unfortunately, these intra-Ag-generated immune responses cannot completely eliminate subcutaneous melanoma 28. Therefore, further assessments of the tfRFP-elicited anti-tumor mechanism are required, as is the ability of a tfRFP-elicited specific anti-tumor immune response to be effective during liver metastasis, especially in the liver's maintenance of the immune tolerogenic status.

Intravital imaging is a powerful tool for visualizing tumor immune clearance and molecular events in tumor microenvironments. In a previous study, we used intravital imaging to establish the spatial distribution and migratory behavior of adoptive cytotoxic T lymphocytes (CTLs) and endogenous CTLs in the tumor microenvironment during adoptive cell immunotherapy in melanoma 29. In another investigation, we performed intravital molecular imaging to reveal the restrained capacity of CTLs to kill tumor cells in the liver through dynamically monitoring the fluorescence resonance energy transfer (FRET) signals of caspase-3 and calcium sensors in tumor cells 30. Additionally, for long-term intravital imaging of the liver, we developed a drawer-type abdominal window with an acrylic/resin coverslip named DAWarc, which we used to observe the colonization and growth of liver metastasis for up to 10 days 31. We expect intravital imaging to represent a powerful tool for further evaluating the tfRFP-elicited anti-tumor mechanism, especially its stimulation of specific humoral immune responses in the liver.

In this study, we aimed to verify whether the metastasis formed by tumor cells expressing intra-Ags in the liver could be impeded by the intra-Ag-elicited immune response. We also explored the potential mechanism of the release of intra-Ags at an early stage to trigger an immunologic effect. We hypothesized that ROS levels increase in circulating tumor cells at the early stage of tumor cell entry into the liver and induce slight caspase-3 activation, which cleaves GSDME proteins to punch holes in the tumor cell membrane. The increased permeability of the tumor cell membrane leads to the release of intra-Ags, creating the possibility of a liaison between the Ags and antibodies in the tumor microenvironment. The formation of intra-Ag-antibody immune complexes initiates complement activation and forms MACs, which further accelerates the release of intra-Ags from tumor cells. Surprisingly, our results indicated that the tfRFP-elicited immune responses completely inhibited the metastasis of tfRFP-expressing melanoma cells in the liver. Our findings also showed that intra-Ag-antibody immune complexes, the complement system, neutrophils, and macrophages played critical roles in tumor clearance by intra-Ag-elicited humoral immune responses generated through the ROS-caspase-3-GSDME pathway.

## Results

### The tfRFP-elicited immune responses inhibited tfRFP-expressing melanoma cell metastasis in the liver

**Figure 1:**
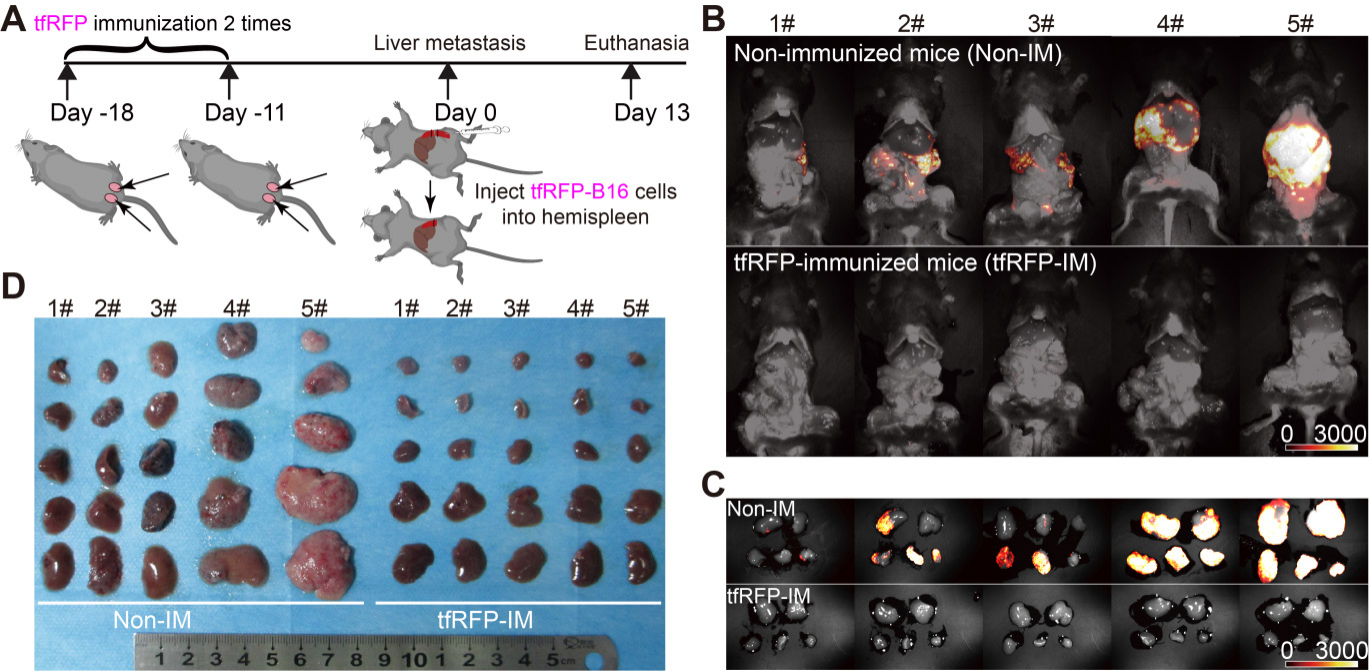
**The tfRFP-elicited immune responses inhibited tfRFP-expressing melanoma cell metastases in the liver.** (**A**) Schedules of tfRFP immunization and the generation of the liver metastasis model. (**B**) Representative whole-body fluorescence imaging of tfRFP-immunized and non-immunized mice. (**C**) Representative wide-field fluorescence imaging of 5 lobes of the liver in tfRFP-immunized and non-immunized mice. (**D**) Representative white-light images of 5 lobes of livers in tfRFP-immunized and non-immunized mice. Data are from 3 replicate experiments, with n = 15 per group.

The liver has an immune tolerogenic microenvironment. Therefore, we assessed whether the tfRFP-induced specific anti-tumor immune response remains effective during tumor metastasis in the liver. Mice were immunized with a mixture of purified tfRFP antigen and incomplete Freund's adjuvant on two sides of the tail base (50 μg tfRFP per side) through two subcutaneous injections (at 18 days and 11 days before tumor cell injection, one-week interval) and then were inoculated with 1 × 10^6^ tfRFP-B16 cells into the hemispleen (defined as Day 0) to generate the liver metastasis model (Figure 1A). Thirteen days after inoculation with tfRFP-B16 tumor cells, the mice were euthanized, and their livers were exposed for imaging. Whole-body fluorescent imaging revealed that the livers of mice in the non-immunized group expressed strong tfRFP signals, and almost no tfRFP signal appeared in the tfRFP-immunized group (Figure 1B).

To clearly observe metastases in the liver, we extracted the livers for wide-field fluorescence imaging (Figure 1C) and white-light imaging (Figure 1D). The results showed that tfRFP-B16 metastasized in all the liver lobes of non-immunized mice, and the volumes of liver lobes with metastases were significantly larger (Figure 1D). All the liver lobes in the tfRFP-immunized group did not exhibit tfRFP signals and maintained normal liver shapes and volumes (Figure 1D). HE (hematoxylin and eosin) staining sections of the livers also hardly detected any liver metastasis but displayed inflammatory cell infiltration in the tfRFP-immunized mice 13 days after tfRFP-B16 cells injection (Figure S1A-C). However, in the non-immunized mice on day 13 after tfRFP-B16 cells injection, there were an average of 50 metastatic sites and 35.3% of the area was occupied by the tumor tissue in the HE staining sections (Figure S1A-C). These data suggested that the tfRFP-elicited immune responses efficiently inhibited tfRFP-B16 metastasis in the livers of tfRFP-immunized mice.

### Intravital fluorescent imaging revealed the experience of tfRFP-B16 tumor cells in the liver

**Figure 2:**
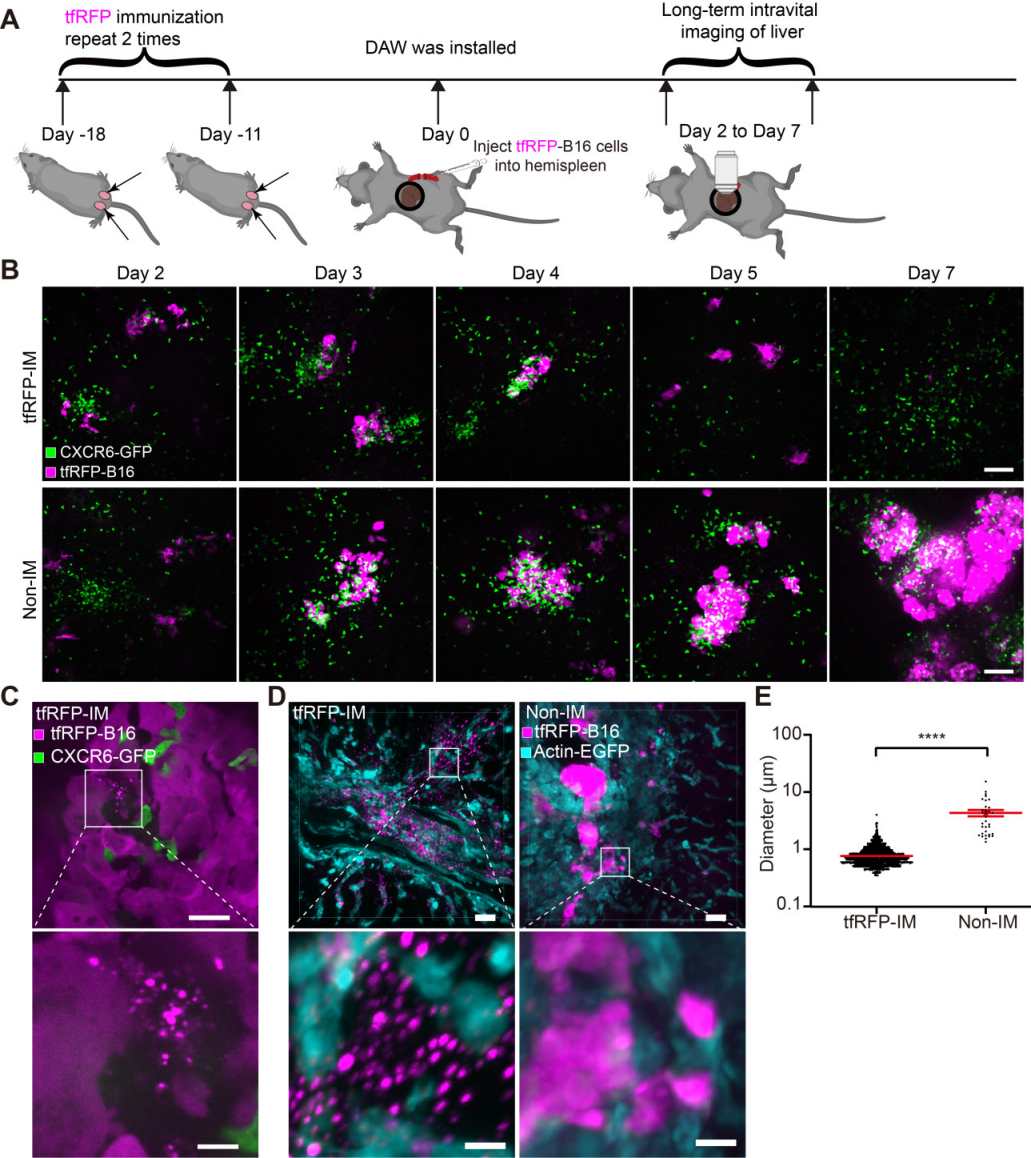
**Intravital fluorescence imaging revealed the experiences of tfRFP-B16 tumor cells in the liver.** (**A**) Schedules for long-term intravital imaging of liver metastasis, in which a DAW was installed on the day that tfRFP-B16 cells were injected into the liver *via* the spleen. (**B**) Representative long-term intravital imaging of tfRFP-B16 metastases in the livers of CXCR6^GFP/+^ mice from Day 2 to Day 7. The images are from a tfRFP-immunized mouse (top row) and a non-immunized mouse (bottom row). Scale bar, 100 µm. (**C**) A representative image of infiltrated CXCR6-GFP cells and tfRFP^+^ microparticles in tfRFP-B16 metastasis from tfRFP-immunized mice on Day 3. Scale bar in the large image, 20 µm. Scale bar in the enlarged image, 5 µm. (**D**) Representative confocal imaging of liver tissues from tfRFP-immunized and non-immunized mice on Day 3. The characteristics of the morphology of tfRFP-B16 cells in the liver differed between the tfRFP-immunized and non-immunized groups. Scale bar in the large image, 20 µm. Scale bar in the enlarged image, 5 µm. (**E**) The diameter of tfRFP^+^ particles from the tfRFP-immunized and the non-immunized groups. Each data point represents a single tfRFP^+^ particle. Data are presented as mean ± SEM. Statistical analysis was performed using the Mann Whitney test, ****P < 0.0001.

To determine the order of occurrence between liver metastasis and elimination by the tfRFP-elicited immune responses, we performed long-term intravital imaging of the liver using a drawer-type abdominal window (DAW), from Day 2 to Day 7 after tfRFP-B16 cells injection into tfRFP-immunized and non-immunized mice, and used HE staining to evaluate the liver metastasis (Figure 2A and Figure S2). CXCR6^GFP/+^ mice were used, and the GFP signal represents the infiltration of immune cells, including iNKT cells (~70%), CD4^+^ T cells (2.40%), CD8^+^ T cells (3.67%), and NK cells (21.48%) (Figure S3A-B).

Imaging data revealed that tfRFP-B16 cells colonized the liver in the first several days and were eliminated from the liver of tfRFP-immunized mice (top row in Figure 2B). A considerable number of CXCR6-GFP cells were recruited to the liver and accumulated around the tumor in both tfRFP-immunized and non-immunized mice (Figure 2B). Flow cytometry results showed that the percentage of infiltrated iNKT cells in the CXCR6-GFP cells of the liver has no significant difference between the tfRFP-immunized and non-immunized groups, except on Day 4 (Figure S3B-C). We further examined the expression of Ki67 in iNKT cells, CD8^+^ T cells and CD4^+^ T cells on Day 4, and the expression of IFN-γ in iNKT cells, NK cells, CD8^+^ T cells, and CD4^+^ T cells on Day 3 in the livers of tfRFP-immunized and non-immunized mice. Although there was no significant difference between the two groups, the expression of Ki67 and IFN-γ in those immune cells in the tfRFP-immunized group still had an increasing trend compared with the non-immunized mice (Figure S3D-E).

To further confirm the role of iNKT cells in the tfRFP-induced immune response to liver metastasis, iNKT cell knockout (Jα18^-/-^) mice were immunized twice with tfRFP and then inoculated with tfRFP-B16 cells *via* the spleen. According to our findings, tfRFP-B16 cells remained cleared in the iNKT cell knockout mice after tfRFP immunization (Figure S4A-B). These data suggest that iNKT cells are not the major effector cells in tfRFP-elicited anti-tumor immune responses.

High-resolution intravital imaging uncovered the production of tfRFP^+^ microparticles during metastasis in the liver of tfRFP-immunized mice on Day 3 (Figure 2C, Movie S1). To examine whether the tfRFP^+^ microparticles were specifically produced in the tfRFP-immunized mice, we observed the liver tissues from the tfRFP-immunized and non-immunized actin-EGFP mice 3 days after tfRFP-B16 cells injection (Figure 2D). In the tfRFP-immunized mice, tfRFP^+^ microparticles (mean diameter was 0.75 ± 0.01 μm) were generated near the tfRFP-B16 tumor cells, and in the non-immunized mice, the larger tfRFP^+^ particles (mean diameter was 4.18 ± 0.54 μm) were observed in the tfRFP-B16 tumor area (Figure 2D-E).

### tfRFP immunization induced a specific humoral immune response effect on tumor cells expressing the intracellular tfRFP antigen in the hepatic sinusoid

**Figure 3:**
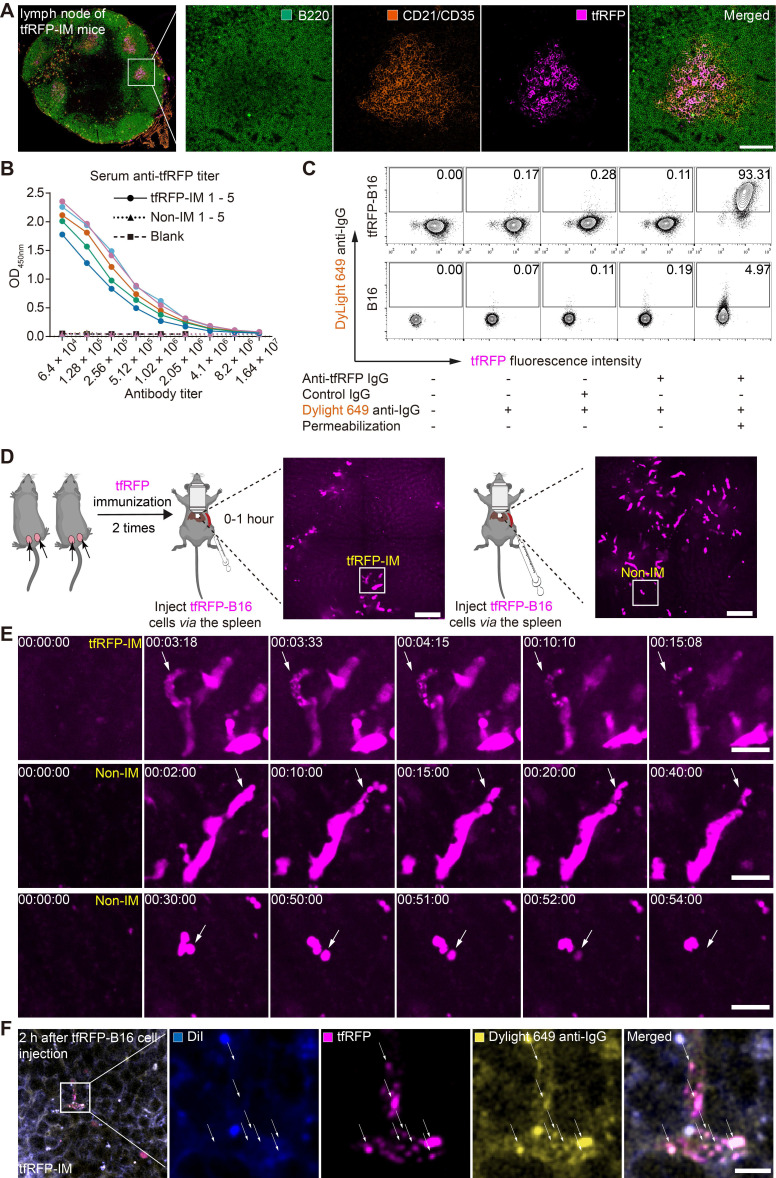
** The tfRFP-elicited humoral immune response leads to the formation of intra-Ag-antibody complexes at the early stage of tfRFP-B16 circulation into the liver.** (**A**) Representative immunofluorescence images of tfRFP antigen uptake by CD21/CD35^+^ fDC cells in the inguinal lymph nodes of tfRFP-immunized mice. Scale bar, 100 µm. (**B**) The titer curve for anti-tfRFP specific antibodies from the serum of tfRFP-immunized or non-immunized mice. Representative profiles are from 5 biological replicates. (**C**) A representative flow cytometry plot for tfRFP-B16 cells labeled with anti-tfRFP IgG with or without permeabilization. (**D**) Representative intravital images of tfRFP-B16 tumors during tumor cell circulation into the liver. Scale bar, 100 µm. (**E**) Representative intravital images of the disruption of tfRFP-B16 cells to form tfRFP^+^ microparticles within the first hour after injecting tfRFP-B16 tumor cells into the spleen of tfRFP-immunized mice (top row); representative intravital images of the formation of tfRFP^+^ particles in the liver of non-immunized mice (middle row); disruption in the non-immunized mice occurred with tfRFP disappearance (bottom row). Scale bar, 30 µm. (**F**) A representative immunofluorescence image of liver tissues at the first hour of tumor inoculation, showing IgG positive tfRFP^+^ microparticles in the tfRFP-immunized group. Scale bar, 10 µm.

Because the tfRFP-elicited immune responses successfully eliminated tfRFP-B16 metastasis in the liver in the early days post-tumor cell inoculation and iNKT cells were not the major effector cells, we examined whether tfRFP-elicited humoral immunity played an integral role in the elimination of tfRFP-B16 tumor cells in the liver. First, we extracted the inguinal lymph nodes from tfRFP-immunized mice for 3D imaging (Figure S5A-B) and immunofluorescence staining analysis (Figure 3A and Figure S5C). The results showed that tfRFP was stored in the germinal centers of lymph nodes and taken up by follicular dendritic cells (fDCs, CD21^+^CD35^+^ labeled), in which the fluorescence signal of tfRFP was still maintained (Figure 3A and Figure S5C).

Reportedly, fDCs continuously stimulate B cells in the germinal center to produce antibodies 32. The titer of anti-tfRFP IgG in serum was identified using ELISA (enzyme-linked immunosorbent assay). As the data in Figure 3B show, after two immunizations, the anti-tfRFP IgG in the serum was detectable even after 4 million dilutions (Figure 3B), indicating that the titer of anti-tfRFP IgG in tfRFP-immunized mouse serum was about 1:4,000,000 18 days after the first immunization.

To determine whether tfRFP is an intracellular or extracellular antigen in tfRFP-B16 cells, anti-tfRFP IgG was purified from the serum of tfRFP-immunized mice and incubated with tfRFP-B16 cells for 30 mins at 4 °C, which were treated with or without a membrane permeabilization reagent. The DyLight 649 goat anti-mouse IgG antibody (DyLight 649 anti-IgG) was used as the secondary antibody for flow cytometry analysis. DyLight 649 signals were barely detected in tfRFP-B16 cells without membrane permeabilization. In contrast, most tfRFP-B16 cells displayed strong DyLight 649 signals after membrane permeabilization (Figure 3C). These results indicate that tfRFP is an intracellular antigen in tfRFP-B16 tumor cells and is only expressed inside but not on the membrane of tfRFP-B16 cells.

To observe the different experiences of tfRFP-B16 cells circulating into the livers of tfRFP-immunized mice and non-immunized mice, time-lapse intravital imaging was performed soon after tfRFP-B16 injection *via* the spleen (Figure 3D). Imaging data showed that in the tfRFP-immunized mice, some tfRFP-B16 cells were disrupted to generate tfRFP^+^ microparticles (diameter about 0.75 μm) (top row in Figure 3E, Movie S2). In the non-immunized mice, some tfRFP-B16 cells produced membrane-coated tfRFP^+^ particles (diameter about 4 μm) (middle row in Figure 3E, Figure S6 and Movie S3), and in some other tfRFP-B16 cells, the signal of tfRFP disappeared without forming tfRFP^+^ particles when the tfRFP-B16 cells were disrupted (bottom row in Figure 3E, Movie S4). These results suggested that tfRFP-B16 tumor cells circulating into the livers of tfRFP-immunized and non-immunized mice underwent different experiences.

In our previous research, tfRFP^+^ microparticles in the subcutaneous tfRFP-B16 tumor microenvironment of the tfRFP-immunized group formed antigen-antibody complexes 28. Here, we needed to confirm that tfRFP^+^ microparticles in the liver of tfRFP-immunized mice also formed antigen-antibody complexes. tfRFP^+^ microparticles colocalized with DyLight 649 anti-IgG (yellow in Figure 3F) but not with the membrane dye DiI (Ex: 551 nm, Em: 566 nm, blue in Figure 3F). To further verify that tfRFP^+^ microparticles were antigen-antibody complexes, we performed flow cytometry to detect the tfRFP^+^ microparticles produced by *in vitro* cultured tfRFP-B16 cells treated with melittin (a kind of cytotoxic peptide used to punch holes in the cell membrane) and anti-tfRFP IgG. The flow cytometry data showed that tfRFP^+^ microparticles could be labeled with DyLight488 anti-IgG antibody but not with the membrane dye DiR (Ex: 748 nm, Em: 780 nm, Figure S7A-C). The results confirmed that the tfRFP^+^ microparticles contained anti-tfRFP IgG but no membrane of tfRFP-B16 cells and the tfRFP^+^ microparticles formed by tfRFP-B16 cells were antigen-antibody complexes but not other complexes. Now, the question of what trigger the release of intracellular antigens and led to the formation of antigen-antibody complexes must be answered, as is that of how anti-tfRFP IgG affect circulating tumor cells and eliminates them in the liver.

### Some circulating tumor cells in the liver displayed elevated ROS and activated caspase-3, even in the non-immunized mice

**Figure 4:**
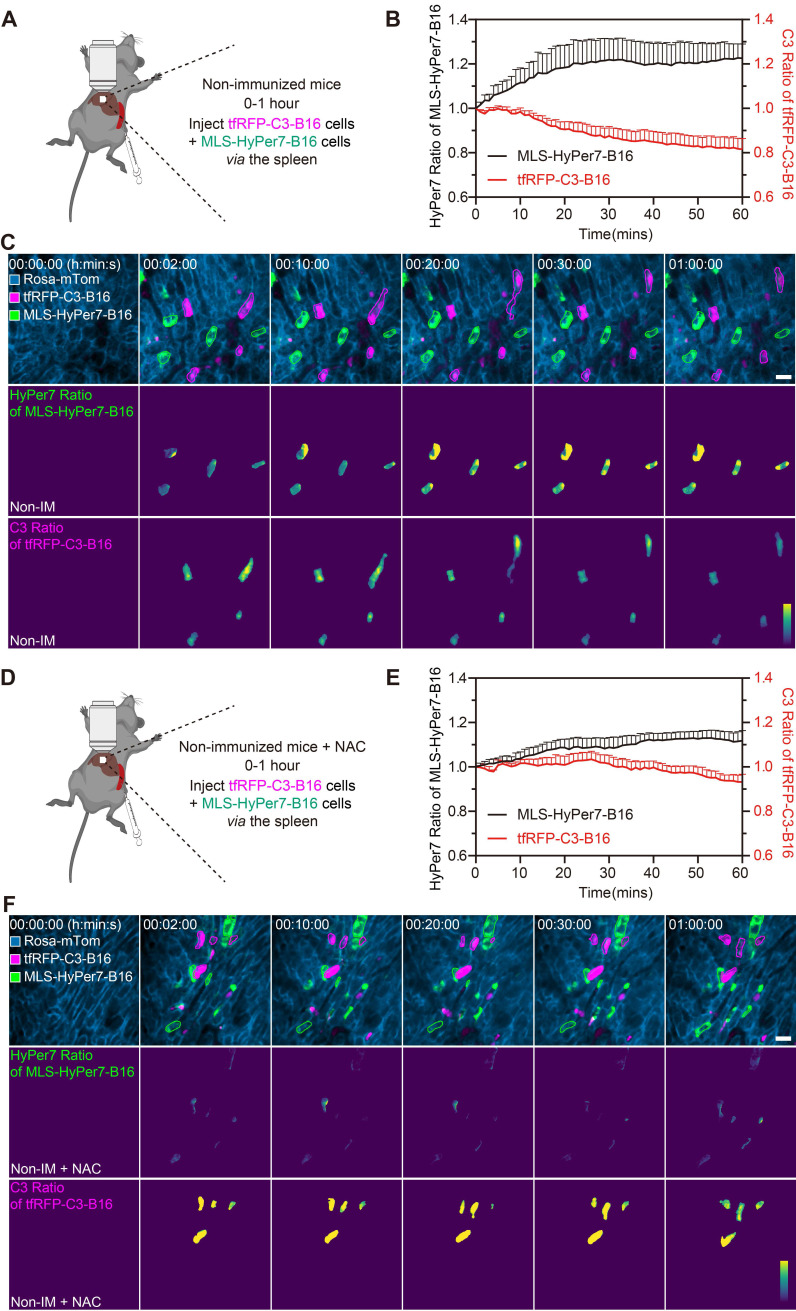
** ROS elevation and caspase-3 activation in tumor cells circulating into the liver.** (**A**) Diagram depicting intravital imaging and (**B**) the change curve for the HyPer7 ratio in MLS-HyPer7-B16 cells (dark curve) and the C3 ratio in tfRFP-C3-B16 cells (red curve). Traces represent mean ± SEM from 3 biological replicates with at least 6 cells per replicate. (**C**) Representative intravital images of the liver in the non-immunized mice; the top row is fluorescence images, the middle row is the HyPer7 ratio (F488/F405) images of MLS-HyPer7-B16 cells, and the bottom row is the C3 ratio (FRET/CFP) images of tfRFP-C3-B16 cells. Scale bar, 20 µm. (**D**) Diagram depicting intravital imaging and (**E**) the change curve for the HyPer7 ratio in MLS-HyPer7-B16 cells (dark curve) and the C3 ratio in tfRFP-C3-B16 cells (red curve). Traces represent the mean ± SEM from 3 biological replicates with at least 6 cells per replicate. (**F**) Representative intravital images of the liver in the non-immunized mice injected with NAC. Scale bar, 20 µm.

Reportedly, ROS in circulating tumor cells increases compared to ROS in tumor cells grown *in situ* 33, 34. To assess ROS levels in circulating tfRFP-B16 cells, especially those passing through hepatic sinusoids under fluid shear stress from blood flow 35, we screened B16 tumor cells stably expressing the mitochondria localized-ROS probe (MLS-HyPer7-B16 cells) and tfRFP-B16 tumor cells stably expressing the caspase-3 probe (tfRFP-C3-B16 cells). First, we performed intravital imaging of the livers in the non-immunized mice soon after injecting tumor cells (a mixture of a 1:1 ratio of MLS-HyPer7-B16 cells and tfRFP-C3-B16 cells) *via* the spleen (Figure 4A). The MLS-HyPer7-B16 cells displayed elevated ROS signals several minutes after tumor cell inoculation, reaching peak levels after about 20 mins (Figure 4B-C). In addition, caspase-3 was activated in some tfRFP-C3-B16 cells with decreased C3 ratio (detailed calculation method is in the Methods section) about 10 mins after tumor cell inoculation, maintaining the sustained activation for over 30 mins (Figure 4B-C and Movie S5).

Next, the ROS inhibitor NAC (N-acetyl-L-cysteine) was used to test whether inhibiting ROS could prevent the caspase-3 activation in circulating tumor cells (Figure 4D). Intravital imaging revealed that NAC noticeably subdued the ROS signal and caspase-3 activation in tumor cells, with the ROS signal rising slowly and mild caspase-3 activation beginning about 30 mins after tumor cells injection (Figure 4E-F and Movie S6. Elevated ROS in circulating tumor cells should, therefore, be the key factor in inducing caspase-3 activation, and this situation typically exists when tumor cells circulate into hepatic sinusoids, even in normal physiological conditions. All these results indicate that ROS elevation and caspase-3 activation are possibly crucial factors for the release of intracellular tfRFP antigens during the first hour of tumor cell circulation into the liver.

### Elevated ROS is a key factor for punching holes in the cell membrane and releasing intracellular tfRFP antigens

**Figure 5:**
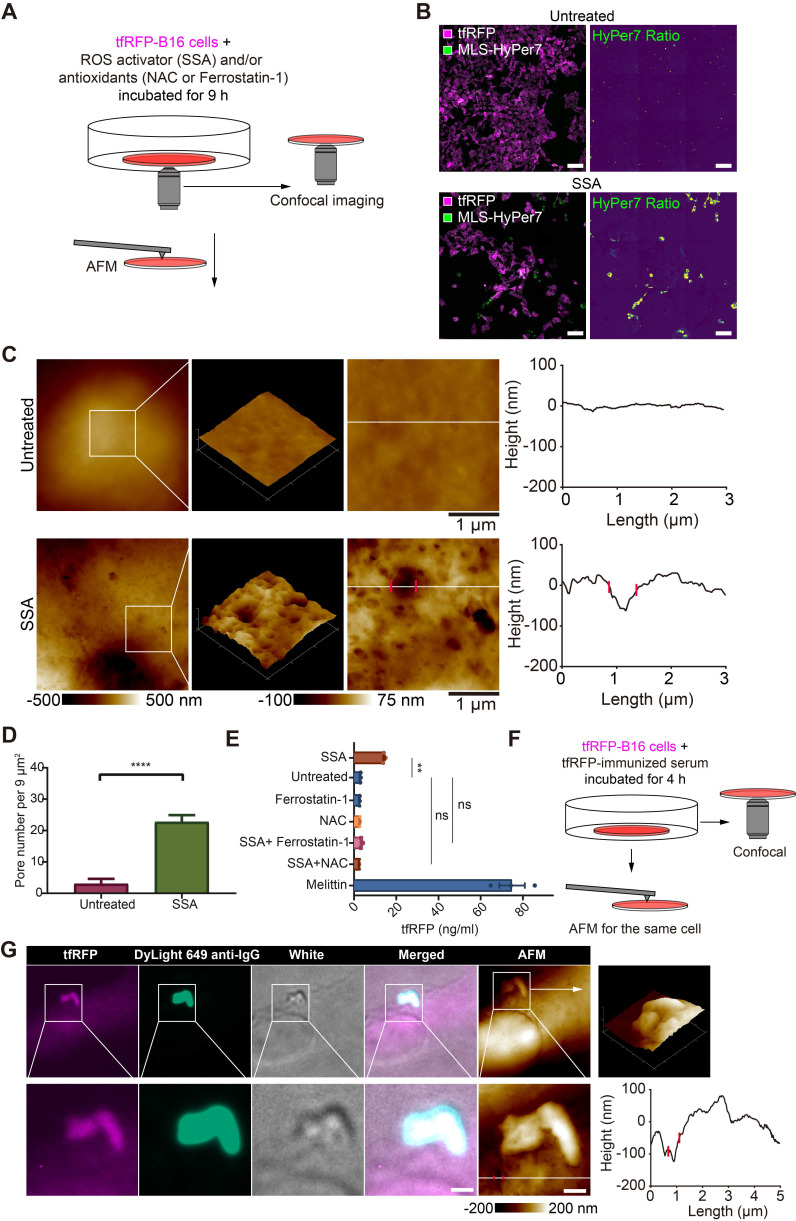
**ROS is a key triggering factor for punching holes in cell membranes and releasing intracellular tfRFP antigens.** (**A**) Diagram depicting confocal imaging and atomic force microscopy (AFM) imaging of tfRFP-B16 cells *in vitro*. (**B**) Representative confocal imaging of ROS signals in MLS-HyPer7-B16 cells treated with or without SSA for 9 hours. Scale bar, 100 µm. (**C**) AFM scan of the cell membrane. The white lines across the pore indicate topographies, the red dots mark the edges of the holes, and the right image depicts the height curve corresponding to the white lines. Scale bar, 1 µm. (**D**) Density statistics of pores on the cell membranes. Data are presented as mean ± SEM. Statistical analysis was performed using the unpaired Student's *t*-test, ****P < 0.0001. (**E**) Histogram of the release of tfRFP from cultured tfRFP-B16 cells. Data are presented as mean ± SEM. Statistical analysis was performed using the Brown-Forsythe and Welch ANOVA tests, ns: not significant, **P < 0.01. (**F**) Diagram depicting immunofluorescence confocal imaging and atomic force scanning imaging of tfRFP-B16 cells treated with tfRFP-immunized serum for 4 hours. (**G**) Representative immunofluorescence confocal images and AFM images of the same cells stained with the DyLight 649 anti-mouse IgG antibody. The white lines across the pore indicate topographies, the red dots mark the edges of the holes, and the right image depicts the height curve corresponding to the white lines. Scale bar, 1 µm.

Given the above speculation, we explored the relationship between ROS elevation and cell punching *in vitro* using tfRFP-B16 cells. tfRFP-B16 cells were treated with an ROS activator (Sulfasalazine, SSA) for 9 hours in a cell-culture dish (Figure 5A). Confocal imaging substantiated the SSA-induced ROS elevation in tumor cells (Figure 5B).

To analyze the ability of elevated ROS to induce punching holes in cell membranes, we scrutinized the cell membrane using atomic force microscopy (AFM). The obtained AFM images bore many recognizable punched holes in the membranes of SSA-treated cells (Figure 5C). The number of punched holes in the SSA-treated cells was 7.58-fold higher than that in the untreated cells (Figure 5D).

The supernatants of tfRFP-B16 cells treated with the ROS activator (SSA) and/or the ROS inhibitor (NAC, or Ferrostatin-1) for 24 hours were examined for the presence of tfRFP antigens using ELISA. Melittin, a membrane-permeabilizing peptide, was used as the positive control. The data showed that tfRFP antigen concentrations in the SSA group (15.13 ng/mL) increased 4.66 times compared to the untreated group (3.25 ng/mL). The release of tfRFP antigen in the SSA + NAC group (2.78 ng/mL) and the SSA + Ferrostatin-1 group (3.78 ng/mL) did not rise significantly (Figure 5E). Remarkably, the anti-tfRFP serum was added to the culture medium (Figure 5F) and tfRFP-B16 cells were stained with DyLight 649 anti-IgG. The confocal and AFM images of the same region of tfRFP-B16 cells displayed the appearance of tfRFP^+^ microparticles and the DyLight 649 anti-IgG signal near a punched hole of the cell membrane in a scene (Figure 5G and Figure S8). Immunofluorescence and AFM findings were consistent, indicating that antigen-antibody complexes formed upon the release of antigens from the cell membrane. In brief, these results point to elevated ROS in tumor cells causing an increase in membrane permeability and the release of intracellular antigens.

### Gasdermin E is a key molecule in caspase-3 activation-induced cell membrane punching

**Figure 6:**
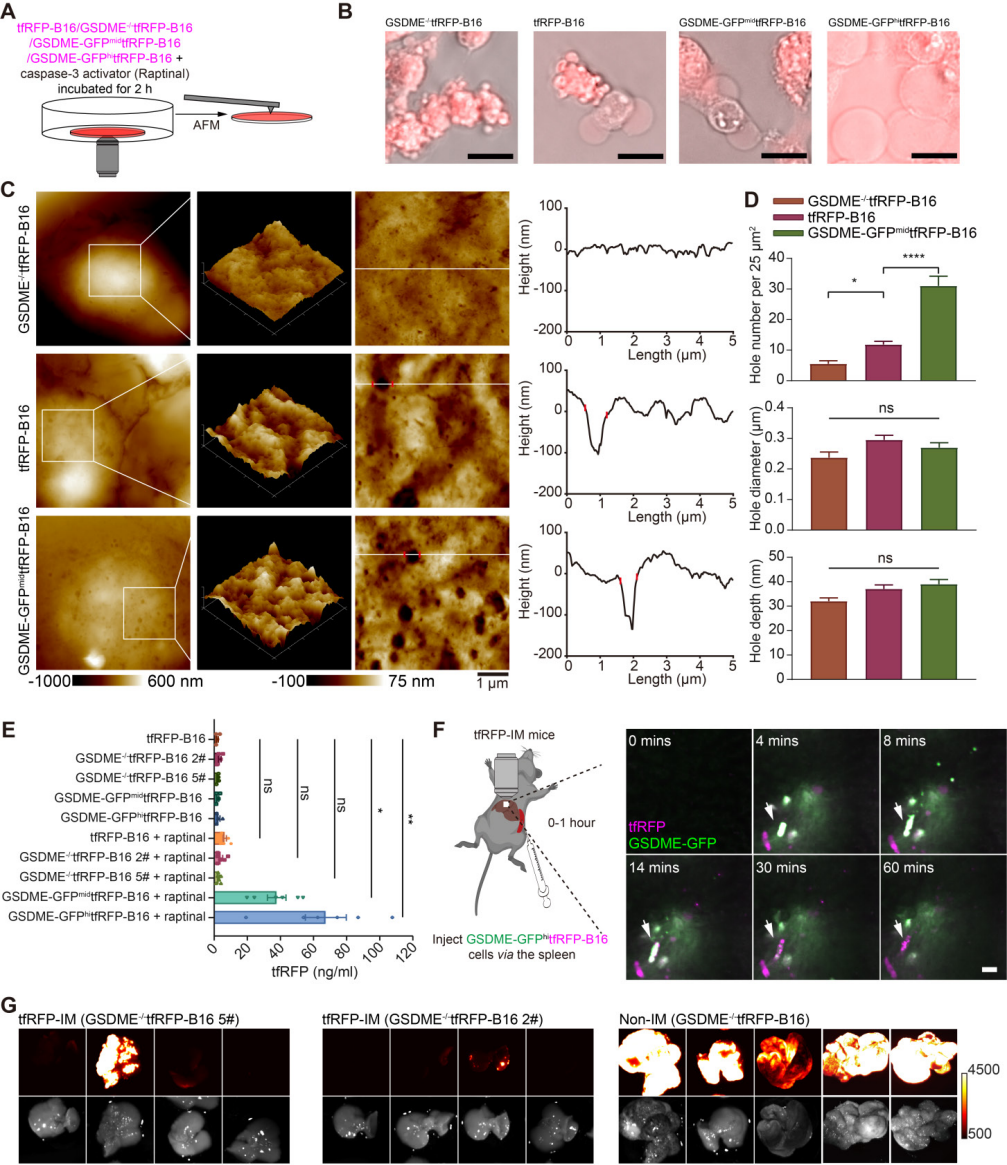
**GSDME is a key molecule in caspase-3 activation induced cell membrane punching.** (**A**) Diagram depicting microscopic imaging and atomic force scanning imaging. (**B**) Representative microscopic images of cell morphology and tfRFP fluorescence. Scale bar, 20 µm. (**C**) AFM scan of the cell membrane. The white lines across the pore indicate topographies, the red dots mark the edges of the holes, and the right image depicts the height curve corresponding to the white lines. Scale bar, 1 µm. (**D**) The assessed density, depth, and diameter of holes in three groups of tfRFP-B16 cells expressing different levels of GSDME. (**E**) ELISA evaluation of the release of tfRFP from cultured tfRFP-B16 cells treated with Raptinal for 2 hours. Statistical analysis was performed using the one-way ANOVA with Tukey's multiple comparisons post-test or the Kruskal-Wallis test followed by Dunn's multiple comparison tests, ns: not significant, *P < 0.05, **P < 0.01. (**F**) Representative intravital imaging and time-lapse images of the liver within one hour after injecting tfRFP-B16 tumor cells into the spleen of tfRFP-immunized mice. Scale bar, 20 µm. (**G**) Representative wide-field fluorescence images of livers on day 13 after GSDME^-/-^tfRFP-B16 cell inoculation. Data are from 2 replicate experiments, with n = 5~8 per group.

Activated caspase-3 can cleave gasdermin E (GSDME) to GSDME-N, perforating the cell membrane from the inside 16. Hence, we speculated that caspase-3 activation in circulating tfRFP-B16 cells could cleave GSDME to promote cell membrane punching. To verify this speculation, we screened tfRFP-B16 tumor cells with moderate or high expression of GSDME with green fluorescence protein (GFP) (Figure S9A) or without GSDME expression (Figure S9B), grouped as GSDME-GFP^mid^tfRFP-B16 cells, GSDME-GFP^hi^tfRFP-B16 cells, and GSDME^-/-^tfRFP-B16 cells, respectively. Tumor cells were treated with a caspase-3 activator (Raptinal) for 2 hours to induce caspase-3 activation *in vitro* (Figure 6A). Fluorescence microscopic imaging of Raptinal-treated cells showed that GSDME^-/-^tfRFP-B16 cells had apoptotic features with strong tfRFP signals, tfRFP-B16 cells and GSDME-GFP^mid^tfRFP-B16 cells displayed both apoptotic and pyroptotic characteristics, while GSDME-GFP^hi^tfRFP-B16 cells exhibited pyroptotic features with significant leakage of tfRFP antigens into the medium (Figure 6B). AFM images revealed numerous punched holes in the membranes of Raptinal-treated GSDME-GFP^mid^tfRFP-B16 cells (Figure 6C). The number of punched holes in GSDME-GFP^mid^tfRFP-B16 cells was 2.63-fold and 5.64-fold higher than those in tfRFP-B16 cells and GSDME^-/-^tfRFP-B16 cells, respectively (Figure 6D). The ELISA detection of the concentrations of tfRFP antigen released in cell culture supernatants showed that the release of tfRFP antigen in Raptinal-treated GSDME-GFP^hi^tfRFP-B16 cells was 10.53-fold higher than that of tfRFP-B16 cells and 19.74-fold higher than that of GSDME^-/-^tfRFP-B16 cells (Figure 6E). These data suggest that the caspase-3 activation inducing the release of tfRFP antigens is due to GSDME-mediated membrane punching.

Intravital imaging further confirmed that circulating GSDME-GFP^hi^tfRFP-B16 cells in the livers of tfRFP-immunized mice were rapidly disrupted, accompanied by the formation of tfRFP^+^ microparticles and the loss of the GSDME-GFP signal (Figure 6F and Movie S7). Furthermore, using GSDME^-/-^tfRFP-B16 cells to generate liver metastasis models demonstrated that knocking out GSDME from tfRFP-B16 cells resulted in partial recovery of the colonization ability of tfRFP-B16 cells in the livers of tfRFP-immunized mice (Figure 6G). This finding suggests that caspase-3 activation promotes the cleavage of GSDME and that GSDME-N-mediated membrane punching is a key step in the release of the intracellular antigen tfRFP and the formation of antigen-antibody complexes.

### Immune complexes on the tumor cell membrane activated the complement system to form MACs, promoting the generation of more immune complexes

**Figure 7:**
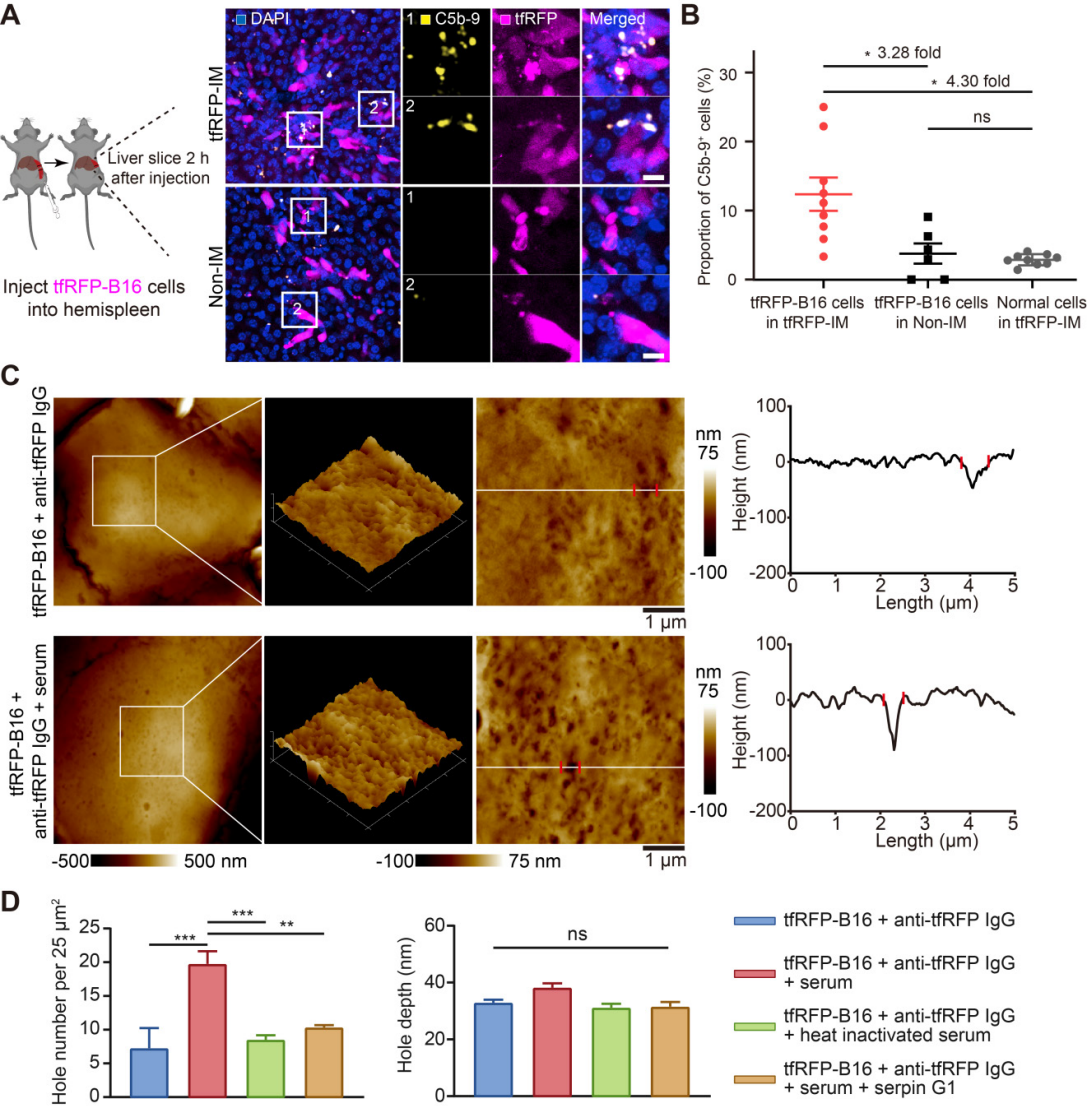
**Immune complexes activated the complement system to amplify cell punching.** (**A**) Representative immunofluorescence images of C5b-9 in the liver 2 hours after tumor cell injection. Scale bar, 10 µm. (**B**) Proportion of C5b-9^+^ cells in the tfRFP-B16 cells or normal cells (except for tfRFP-B16 cells). Each dot represents the proportion of cells with a C5b-9^+^ signal in all corresponding cells in a field of liver sections from three mice. Data are presented as mean ± SEM. Statistical analysis was performed using the Brown-Forsythe and Welch ANOVA tests, ns: not significant, *P < 0.05. (**C**) Representative AFM images of tfRFP-B16 cells after 4 hours of treatment with anti-tfRFP IgG + normal mouse serum or only anti-tfRFP IgG. The white lines across the pore indicate topographies, the red dots mark the edges of the holes, and the right image depicts the height curve corresponding to the white lines. Scale bar, 1 µm. (**D**) The assessed density and depth of holes for comparison of the holes of tfRFP-B16 cells treated with anti-tfRFP IgG without normal mouse serum, anti-tfRFP IgG + normal mouse serum, anti-tfRFP IgG + heat-inactivated mouse serum, and anti-tfRFP IgG + normal mouse serum + complement C1 inhibitor Serpin G1. Statistical analysis was performed using the one-way ANOVA with Tukey's multiple comparisons post-test or the Kruskal-Wallis test followed by Dunn's multiple comparison tests, ns: not significant, **P < 0.01, ***P < 0.001.

The formation of immune complexes on the cell membranes can activate the complement system through classical pathways by binding to complement C1 9, 36. Thus, we speculated that immune complexes could activate the complement system and form MACs in the membranes of tfRFP-B16 cells. We used immunofluorescence imaging of liver tissues to detect the formation of MACs with Alexa Fluor 647 anti-C5b-9 (the marker of MACs) antibody. The data showed that several MACs in the membranes of tfRFP-B16 cells in the tfRFP-immunized mice (Figure 7A and Figure S10), and the proportion of C5b-9^+^ cells in the tfRFP-B16 cells in the tfRFP-immunized mice was 3.28-fold higher than that in the non-immunized mice (Figure 7B). Then, we compared the proportion of C5b-9^+^ cells in the tfRFP-B16 cells with those in the normal cells, and the proportion of C5b-9^+^ cells in the tfRFP-B16 cells was 4.30-fold higher than that of C5b-9^+^ cells in the normal cells in the tfRFP-immunized mice (Figure 7B). Moreover, there was no difference in the proportion of C5b-9^+^ cells between the normal cells in the tfRFP-immunized mice and tfRFP-B16 cells in the non-immunized mice (Figure 7B). These results suggested that in the tfRFP-immunized mice, only the tfRFP-B16 cells were attacked by MACs, and the normal cells were rarely attacked by MACs.

Next, we performed AFM to verify the disruption of cell membranes by activating the complement system through the classical pathways. Complement C1 is a unique component that differentiates the classical pathway of the complement system from the other two pathways (the alternative pathway and the lectin pathway). Therefore, the activation of the classical pathway of the complement system could be inhibited by the C1-specific inhibitor Serpin G1 37, while the other two pathways could not. As the data in Figure 7C-D show, tfRFP-B16 cells treated with purified anti-tfRFP IgG plus normal mouse serum (from non-immunized mouse, containing the complement components) had more pores (about 19 pores per 25 μm^2^) in their membranes than the anti-tfRFP IgG without normal mouse serum group (about 7 pores per 25 μm^2^), the anti-tfRFP IgG with heat-inactivated mouse serum group (about 8 pores per 25 μm^2^) and the anti-tfRFP IgG with normal mouse serum plus complement C1 inhibitor Serpin G1 group (about 10 pores per 25 μm^2^). The above results confirmed that the formation of the tfRFP antigen-antibody complex can activate the complement system through the classical pathway. Compared to tfRFP-B16 cells treated with anti-tfRFP IgG plus heat-inactivated mouse serum, tfRFP-B16 cells treated with anti-tfRFP IgG plus normal mouse serum (containing the complement system) displayed 1.7-fold stronger IgG fluorescence signals on their cell membranes (Figure S11A-B).

These results suggest that antigen-antibody complexes formed on the membranes of tfRFP-B16 cells at the initial stage of tfRFP-B16 circulation into the liver, which activated the complement system through the classical pathway to form MACs in membranes of tfRFP-B16 cells, resulting in punching holes in cell membranes and exposing more intracellular tfRFP antigens.

### Neutrophils were rapidly recruited, and KCs phagocytized dying tfRFP-B16 cells

**Figure 8:**
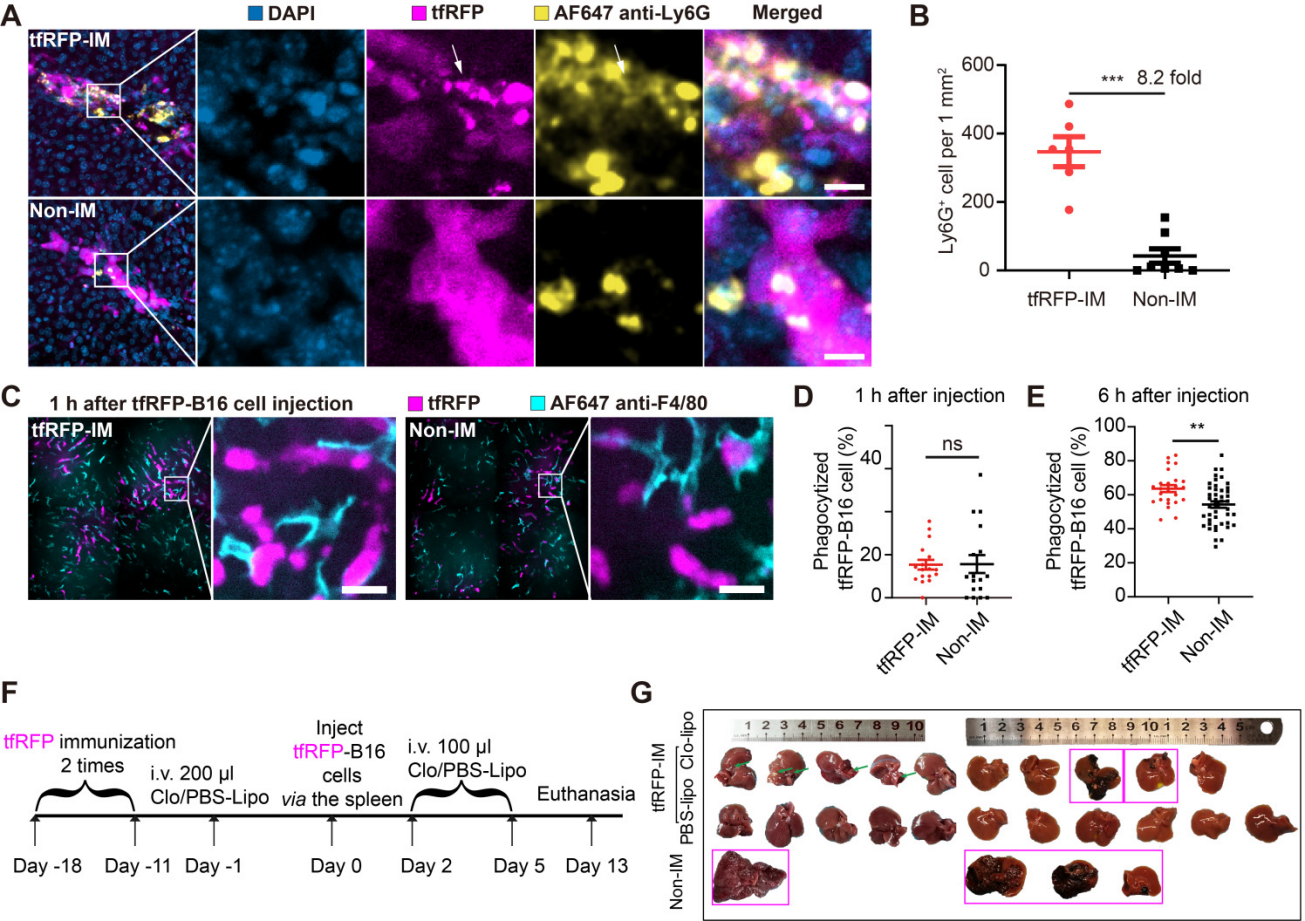
** Recruitment of neutrophils, and phagocytosis functions of macrophages.** (**A**) Representative images of neutrophils (Ly6G^+^) at tfRFP-B16 metastatic loci in tfRFP-immunized and non-immunized mice 2 hours after tfRFP-B16 cell injection. Scale bar, 10 µm. (**B**) The assessed density of neutrophils in the liver sections; each dot represents the density of neutrophils in an image. Data are presented as mean ± SEM. Statistical analysis was performed using the Mann Whitney test, ***P < 0.001. (**C**) Representative intravital images of macrophages (F4/80^+^) at tfRFP-B16 metastasis in tfRFP-immunized and non-immunized groups 1 hour after tfRFP-B16 injection. Scale bar, 20 µm. (**D** and **E**) Statistics of tumor cells phagocytosed by F4/80 macrophages 1 hour (**D**) and 6 hours (**E**) after tfRFP-B16 cells injection. Each dot represents the proportion of tfRFP-B16 cells phagocytosed by F4/80^+^ cells to total tfRFP-B16 cells in a liver section. Data are presented as mean ± SEM. Statistical analysis was performed using the Mann Whitney test, ns: not significant, **P < 0.01. (**F**) Schedules for tfRFP immunization and the generation of the liver metastasis model with KCs depleted using Clodronate-liposomes (**G**) Representative white-light imaging of the liver after euthanasia in each group. Green arrows indicate areas of inflammation, and magenta boxes show livers with tumors. Clo-Lipo, Clodronate-liposomes. PBS-Lipo, PBS-liposomes. Data are from 2 replicate experiments, with n = 4~11 per group.

Because the formation of immune complexes and the activation of the complement system played critical roles in eliminating liver metastasis in the tfRFP-immunized mice, we sought to determine what kind of immune cells participated in the clearance of tfRFP-B16 tumor cells. We performed immunofluorescence imaging of liver tissues 2 hours after tfRFP-B16 cell injection. As shown in Figure 8A, some neutrophils were recruited near tfRFP-B16 tumor cells in the tfRFP-immunized mice, with some neutrophils engulfing tfRFP^+^ microparticles, and the Ly6G^+^ cell number was 8.2-fold higher than that in the non-immunized group (Figure 8B). Meanwhile, few NK cells, CD8^+^ T cells, B cells, dendritic cells, and macrophages infiltrated the tumor (Figure S12A-B). Furthermore, we performed a live cell confocal imaging experiment that tfRFP-B16 cells co-cultured with bone marrow cells (neutrophils: about 60%, B cells: about 20%, monocytes: about 10%, T cells: about 10% 38, 39), which were treated with anti-tfRFP IgG alone or anti-tfRFP IgG plus complement-containing serum for 2 hours. The confocal imaging data showed that in the presence of complement-containing serum, tfRFP-B16 cells were surrounded by more bone marrow cells (most of them were neutrophils, Figure S13A-B). These results suggested that neutrophils were efficiently and quickly recruited to tumor regions as soon as immune complexes were formed and the complement system was activated.

Antibody-dependent cell phagocytosis (ADCP) by macrophages is another antibody-mediated tumor clearance pathway prominent in removing tumor cells with extracellular antigens in the liver 8. However, the role of ADCP in tumor cells with intra-Ags is still not known. Kupffer cells (KCs) are the major tissue-resident macrophages in the liver and are localized in hepatic sinusoids. Intravital imaging revealed the attachment of KCs to tfRFP-B16 cells in both tfRFP-immunized and non-immunized mice 1 hour after tumor cell injection (Figure 8C). However, the proportion of tumor cells phagocytosed was not significantly different between the two groups within 1 hour of injection. (Figure 8D). However, 6 hours after tfRFP-B16 injection, the phagocytic ratio of tfRFP-B16 to KCs in the tfRFP-immunized group (63.64%) was markedly higher than that in the non-immunized group (54.26%, 1.17-fold, *p* < 0.001, Figure 8E). This result suggests that KCs could not directly capture living tfRFP-B16 but demonstrated a stronger phagocytic affinity for dying tfRFP-B16 upon intracellular tfRFP antigen release to the cell membrane and the formation of immune complexes with antibodies.

To test the role of KCs in this model, we deleted KCs using clodronate liposomes (Figure 8F). Our findings showed that one-fifth of tfRFP-immunized mice developed liver metastases in the absence of KCs (Figure 8G), indicating that KCs probably play a fundamental role in the humoral immune response-mediated clearance of tumor cells with intra-Ags in the liver.

## Discussion

By using intravital molecular imaging and the fluorescent intracellular antigen tfRFP, we uncovered how a tfRFP-elicited humoral immune response inhibits tfRFP-B16 metastasis in the livers of tfRFP-immunized mice. This approach enabled us to understand the molecular mechanisms that trigger the release of intra-Ags and the formation of antigen-antibody complexes, the latter being a key step in the activation of the complement system and the recruitment of neutrophils. Although the liver is an immune tolerant organ, the tfRFP-elicited immune responses still completely subdued liver metastasis, which was unaffected by the immune tolerance status of the liver.

It is reported that the tumor cells circulating in the blood vessels have elevated ROS levels due to metabolic changes 33, 34 and shear stress 40. In this study, we found that the formation of intracellular antigen tfRFP-antibody immune complexes during liver metastasis was triggered through the ROS-caspase-3-GSDME pathway in tfRFP-B16 cells. Using intravital imaging and fluorescent protein molecular probes, we observed elevated ROS signals in some circulating tumor cells in the liver of non-immunized mice (Figure 4B-C). We demonstrated that the elevated ROS can act as a trigger for punching holes in tfRFP-B16 tumor cells upon their arrival in hepatic sinusoids (Figure 4B and Figure 4E). An increase in ROS can further activate caspase-3, which cleaves the GSDME protein to GSDME-N 41, resulting in cell membrane perforation (Figure 5C). Moreover, elevated ROS in the tumor cells induces lipid peroxidation and then causes cell membrane damage 42.

In addition to the elevated ROS, there are some triggers that lead to cell membrane being punched, such as mixed-lineage kinase domain-like (MLKL) protein in the necroptosis 22, 43 and perforin released by immune cells (such as NK cells and CD8^+^ T cells) 44. However, unlike the elevated ROS, both MLKL protein-mediated necroptosis and the release of perforin did not occur at the early stage when tumor cells arrived at the liver. In this study, we focused on the molecular events at the early stage of tumor cells circulating into the liver, and the results confirmed that the elevated ROS triggered the subsequent cell punching events.

According to our previous study, the size of tfRFP and bovine serum albumin (BSA) were detected by a fast protein liquid chromatography (FPLC) system 45, and the size of tfRFP is similar to BSA (the diameter is 6~7 nm) 46. Theoretically, the inner diameter of GSDME-N-formed pores 47, 48 might be larger than 10 nm, which allows the release of tfRFP. We also confirmed that the expression level of GSDME in tfRFP-B16 cells positively correlated with the density of holes in the membrane and the release of tfRFP when caspase-3 was activated to cleave GSDME (Figure 6C-E).

In the non-immunized group, when the tfRFP-B16 cells circulated into the liver, the tfRFP-B16 cells underwent three different experiences: 1) some tfRFP-B16 cells released a few membrane-coated tfRFP^+^ particles with an average diameter of 4.18 μm; 2) in some other tfRFP-B16 cells, the signal of tfRFP disappeared when the tfRFP-B16 cells were disrupted (bottom row in Figure 3E, Movie S4); 3) the majority of tfRFP-B16 cells crossed the hepatic sinusoids and colonized the liver in the non-immunized mice. It's reported that tumor cells generate cellular vesicles that contain membrane structures and retain the cytoplasmic fluorophore under the shear stress from blood flow in a lung metastasis model, and these cellular vesicles have an average diameter of 5 μm 49. Their sizes were similar to those of tfRFP^+^ particles (average diameter of 4 μm) in the non-immunized mice. We speculated that the formation of the tfRFP^+^ particles from some tfRFP-B16 cells might be due to the shear stress from blood flow in the hepatic sinusoids. In the second situation, accompanied by the disruption of tfRFP-B16 cells, the fluorescent signal of tfRFP disappeared without forming tfRFP^+^ particles in the non-immunized group (bottom row in Figure 3E and Movie S4). The disruption of tfRFP-B16 cells may also be caused by the shear stress from blood flow in the hepatic sinusoids 23, 50. Although these two situations existed in a small portion of tfRFP-B16 cells, the majority of tfRFP-B16 cells crossed the hepatic sinusoids and colonized the liver (Figure 2B).

In the tfRFP-immunized group, even if a cell membrane was punched a few holes, it still would release the intracellular tfRFP antigens, and then the antigens were quickly captured by anti-tfRFP antibodies in the environment to form immune complexes (Figure 3F and Figure 5G). As we speculated and demonstrated, the formation of immune complexes is a key step in eliciting a series of anti-tumor immune responses, such as activated complement-formed MACs, neutrophil-mediated ADCC, macrophage-involved phagocytosis of dead tumor cells and tfRFP^+^ microparticles, resulting in the complete inhibition of tfRFP-B16 metastasis in the livers of tfRFP-immunized mice. AFM data showed that tfRFP-B16 cells treated with anti-tfRFP IgG plus normal mouse serum (containing the complement) had more pores than cells treated with anti-tfRFP IgG, alone or with heat-inactivated mouse serum, or with normal mouse serum plus complement C1 inhibitor Serpin G1 (Figure 7D), suggesting that tfRFP antigen-antibody complexes on the membrane of tfRFP-B16 cells activated the complement system through the classical pathway to form MACs (Figure 7A and Figure S10), which punched more pores in the membranes of tfRFP-B16 cells. The inner diameters of the complement MACs are about 11 nm 19, which allows the release and exposure of more intracellular tfRFP antigen to bind with anti-tfRFP IgG (Figure S11). Normal cells around the tfRFP-B16 tumor cells do not express tfRFP, which cannot form antigen-antibody complexes with anti-tfRFP IgG on the cell membrane, and they are rarely attacked by MACs (Figure 7B). In the tfRFP-immunized mice, a lot of tfRFP antigen-antibody complexes were formed on the membranes of tfRFP-B16 cells. When some antigen-antibody complexes do not bind to the membranes of tfRFP-B16 cells, most of them are taken up by the immune cells expressing Fcγ receptors, such as neutrophils (Figure 8A), Kupffer cells (Figure 8C) and liver sinusoidal endothelial cells (LSECs) 51. Even if some antigen-antibody complexes nonspecifically adhere to the normal cells, a few complement MACs on the membrane of normal cells are not sufficient to cause cell death 52, 53. In this model, there are two ways of punching holes in the cell membrane, including the elevated ROS triggering the cleavage of GSDME through the ROS-caspase-3-GSDME pathway and the formation of complement MACs. Both ways are necessary and work synergistically to promote the release of intracellular tfRFP antigens. The GSDME-N generated by the cleavage of GSDME through the ROS-caspase-3-GSDME pathway promotes the release of tfRFP antigens and complement MACs formed in the cell membrane play an accelerated role. Complement activation can produce anaphylatoxins (including C3a and C5a), which can cause an inflammatory response and recruit neutrophils 18, 54, possibly explaining why neutrophils but not other immune cells, such as NK cells, CD8^+^ T cells, B cells, dendritic cells and macrophages, were quickly recruited closer to tfRFP-B16 cells in the tfRFP-immunized mice (Figure 8A and Figure S12A-B) and engaged in ADCC to kill tumor cells. We also explored the role of ADCP mediated by KCs (liver resident macrophages) in clearing tfRFP-B16 cells. In the liver, the ADCP of KCs plays a major role in clearing tumor cells expressing extracellular antigens 8. In the tfRFP-immunized mice, the function of KCs is to clean up dying tfRFP-B16 tumor cells and tfRFP^+^ antigen-antibody complexes.

Although the liver is an immune tolerance organ, the humoral immune response induced by the intracellular antigen tfRFP was so effective in clearing tfRFP-B16 metastasis in the liver. The tumor clearing ability of tfRFP-elicited immune responses during metastasis in the liver is apparently higher than that for subcutaneous tumors *in situ* for several possible reasons. First, for reaction time, we speculated that the time required to form antigen-antibody complexes in a liver metastasis model is shorter than that in a subcutaneous tumor model. Tumor cells in the liver metastasis model were injected *via* the spleen, circulated into the liver, and often propagated to hepatic sinusoids. Circulating tumor cells are reportedly metabolically different from subcutaneous clustered tumor cells, including increased levels of ROS 34. Here, we confirmed that elevated ROS in circulating tumor cells is the key triggering factor that induces cell punching and the formation of immune complexes in the liver. Second, although the liver has an inhibitory effect on cellular immune responses 55, 56, for humoral immune responses, the liver harbors more anti-tfRFP IgG and complement components, which are rich in blood, than subcutaneous tumor microenvironments. The liver enhances the possibility of capturing released intracellular antigens, activating complement components and forming MACs. Third, F4/80^+^ macrophages display an important function in eradicating dying tumor cells and immune complexes through Fc receptor-mediated phagocytosis. We also observed that a cleared liver metastasis with disturbed hepatic sinusoid structures was surrounded by dense F4/80^+^ macrophages (Figure S14), including KCs in the liver and monocytes from blood.

Our study revealed that tfRFP-immunization could elicit strong humoral immune responses to eliminate circulating tfRFP-B16 tumor cells in the liver. We also applied the *i.v.* injection of purified anti-tfRFP IgG as a therapy method for tfRFP-B16 metastasis in the liver but did not achieve the expected therapeutic effect, similar to that obtained with tfRFP-antigen pre-immunization. There might be several possible reasons for this failure, such as 1) the dose of the *i.v.* injection of tfRFP IgG was much lower than that of tfRFP immunization-induced antibodies; 2) both LSECs and KCs in the liver express FcγR. For the *i.v.* injection of anti-tfRFP IgG, a significant amount of them will be captured by LSECs and KCs so that the number of anti-tfRFP IgG antibodies targeting tfRFP-B16 tumor cells is greatly reduced. Nevertheless, our research provides a new direction for immunotherapy against intracellular antigens, which is that combination therapy could be performed with tumor vaccine-induced strong humoral immune responses, antibodies targeting intracellular antigens and drugs that promote tumor cell punching.

## Conclusion

In summary, this investigation revealed that intra-Ag-antibody immune complexes, the complement system, neutrophils and macrophages play significant roles during intra-Ag-elicited humoral immune responses, and the ROS-caspase-3-GSDME pathway-induced cell punching is the key step in initiating the antibody recognition of intracellular antigens. Additionally, this research provides a new strategy for tumor immunotherapy through targeting intracellular TAs, which demonstrated a remarkable tumor clearance ability in the liver metastasis.

## Materials and methods

### Mice

C57BL/6 female mice were obtained from Hunan SJA Laboratory Animal Co., Ltd (Hunan, China). B6.129P2-Cxcr6tm1Litt/J (Cxcr6^gfp^, JAX: 005693), B6.129(Cg)-Gt (ROSA) 26Sort^m4(ACTB-tdTomato,-EGFP)Luo^/J (ROSA^mT/mG^, JAX: 007676), and B6.129P-Cx3cr1^tm1Litt^/J (Cx3cr1^EGFP^, JAX: 005582) mice were purchased from the Jackson Laboratory (Bar Harbor, ME, USA). C57BL/6-Tg(CAG-EGFP)/J (actin-EGFP) mice, in which EGFP is expressed in the whole body except erythrocytes and hair, were presented by Prof. Zhiying He (Second Military Medical University, Shanghai, China). All mice were bred and maintained in a specific pathogen-free (SPF) barrier facility at the Animal Center of Wuhan National Laboratory for Optoelectronics. All animal studies were approved by the Hubei Provincial Animal Care and Use Committee and followed the experimental guidelines of the Animal Experimentation Ethics Committee of the Huazhong University of Science and Technology.

### Tumor cells

The B16-F10 cell line was purchased from the BOSTER Company (Wuhan, China). The tfRFP-B16 cell line was established in our lab. The mCerulean3-DEVD-cpVenus (C3) 30, MLS-HyPer7 57, and GSDME-GFP were stably introduced into the B16 or tfRFP-B16 cell lines using the PB transposon system (a gift from Dr. Xiaohui Wu, Fudan University, Shanghai, China) to obtain tfRFP-C3-B16, MLS-HyPer7-B16, MLS-HyPer7-tfRFP-B16, GSDME-GFP^mid^tfRFP-B16 and GSDME-GFP^hi^tfRFP-B16 cells. The GSDME gene of tfRFP-B16 cells was knocked out by the CRISPR-Cas9 gene knockout system to obtain the GSDME^-/-^tfRFP-B16 cell line. The cell morphology and tfRFP fluorescence of GSDME^-/-^tfRFP-B16, tfRFP-B16, GSDME-GFP^mid^tfRFP-B16 and GSDME-GFP^hi^tfRFP-B16 cells were observed by inverted fluorescence microscope (NIB900, Nexcope, Ningbo, China). All cell lines were mycoplasma negative as determined by screening using the MycoProbe Mycoplasma Detection Kit (R&D Systems, Minneapolis, MN, USA). All cell lines mentioned above were cultured in RPMI-1640 (HyClone, Utah, USA) containing 10% FBS (Gibco, New York, USA) and 5% CO_2_ at 37 °C in a constant temperature incubator (Thermo, Massachusetts, USA).

### Plasmid construction

PB-C3 and pRSET-tfRFP were constructed in the previous work of our laboratory. pCS2 MLS-HyPer7 was a gift from Vsevolod Belousov (Addgene plasmid #136470) 57. MLS-HyPer7 was cloned from pCS2+MLS-HyPer7. Then, MLS-HyPer7 was digested by AflII and EcoRI, and cloned into the PiggyBac (PB) vector to obtain PB-MLS-HyPer7. PB-GSDME-GFP was constructed by a ClonExpress MultiS One Step Cloning Kit (Cat# C113, Vazyme, Nanjing, China).

### Protein Purification

KatushkaS158A (tfRFP) were purified as described 28. Briefly, E. coli BL21 (DE3) cells were transformed with pRSET-tfRFP. After full expression, the collected bacteria were frozen at -80 ℃ for at least 30 mins, then thawed and broken by ultrasound. tfRFP was purified by His-Tag Purification Resin (Roche, Basel, Switzerland). The obtained protein was dialyzed in PBS and diluted to a concentration of 2 mg/mL.

### Mouse immunization

Purified tfRFP (2 mg/mL) was mixed with incomplete Freund's adjuvant (IFA) (Sigma, Missouri, USA) in a 1:1 volume ratio. The mixture was vortexed and emulsified for 1 hour by a vortex. Then, 100 μL of the mixture (containing 100 μg tfRFP) was injected into two sides (50 μL for one side) of the tail base of the mouse.

### IgG purification

We collected blood samples from the mice under anesthesia 11-15 days after the second immunization. After clotting, the serum was collected by centrifugation at 8000 g for 5 mins. IgG in serum was purified by Protein A+G Agarose (Beyotime, Shanghai, China). The obtained IgG was exchanged into PBS through a 30 kDa ultrafiltration tube (Amicon Ultra, Germany), and then stored at -20 °C.

### Lymph node optical clearing

The inguinal lymph nodes were optically cleared using the CUBIC method 58 and were infiltrated in the transparent agent for 3D imaging by Zeiss LSM780 (Carl Zeiss MicroImaging, Inc., Jena, Germany). The images were captured using a water 20×/1.0 NA objective.

### Liver metastasis model

Liver metastasis model was established as described 59. Briefly, C57BL/6 mice were anesthetized by i.p. injection with a mixture of 10 mg/kg xylazine and 100 mg/kg ketamine hydrochloride. The spleen was ligated in the middle and divided into two halves, and 1 × 10^6^ tumor cells were injected into the exposed spleen of the mice. Five minutes later, half of the spleen that received the cells was resected to decrease primary tumor growth in the spleen, and the small incision was closed. Body temperature was maintained at 37 °C using a warmer plate (Thermo Plate, TOKAI HIT, Shizuoka-ken, Japan) for recovery until the mouse was mobile and demonstrated regular breathing patterns.

### Measurement of tfRFP concentrations and anti-tfRFP IgG titers

The tfRFP-B16, GSDME^-/-^tfRFP-B16, GSDME-GFP^mid^tfRFP-B16 and GSDME-GFP^hi^tfRFP-B16 cells were seeded in 96-well plates, and the medium was changed before drug treatment. After treatment with the corresponding drug (15 μM Raptinal (Sigma, CAS:1176-09-6), 10 μM Melittin (Apeptide, Shanghai, China), 50 μg purified anti-tfRFP IgG, 500 μM N-Acetyl-L-cysteine (NAC, Sigma, CAS:616-91-1), 500 μM Sulfasalazine (SSA, Sigma, CAS:599-79-1), 2 μM Ferrostatin-1 (Sigma, CAS:1176-09-6), the cell culture supernatant was collected. Mouse-derived anti-tfRFP IgG was diluted to 5 μg/mL with coating buffer (containing 1.59 g/L Na_2_CO_3_ and 2.93 g/L NaHCO_3_ in ddH_2_O_2_). Then 100 μL mouse-derived anti-tfRFP IgG was added to a 96-well ELISA plate (Corning, USA). After overnight incubation at 4 °C, the cells were washed 3 times with PBS-T (PBS containing 0.05% Tween 20) and blocked with PBS-TB (PBS containing 0.05% Tween 20 and 1% BSA) for 2 hours at 37 °C. Then, the PBS-TB solution in the well was discarded. Then, 100 μL of the sample was added to the well plate and incubated for 2 hours at 37 °C. Discard the solution from the well, wash 4 times with PBS-T, add rabbit anti-tfRFP antibody (Cat# AB233, Evrogen, Moscow, Russia), and incubate at 37 °C for 2 h. After washing 4 times with PBS-T, the HRP goat anti-rabbit IgG H&L (Cat# ab97051, Abcam, Cambridge, UK) was added and incubated at 37 °C for 2 h. After washing 5 times with PBS-T, the TMB (Beyotime) was added to the well for 5-10 mins, and the absorption value was measured at 450 nm wavelength and corrected at 620 nm wavelength. Anti-tfRFP IgG titers were measured as reported in a previous article 28.

### Western blot

The 2 × 10^6^ tumor cells were collected in a 1.5 mL EP tube and lysed with 100 μL RIPA lysis solution (Beyotime), in which protease inhibitor cocktail (CWBIO, Beijing, China) was added. The protein concentration was determined by a Detergent Compatible Bradford Protein Assay Kit (Beyotime), and then 30 μg protein samples were run through an SDS-polyacrylamide gel electrophoresis gel and transferred to PVDF membrane blots. Next, the PVDF membrane blots were incubated with Tris buffered saline (TBS) containing 5% nonfat milk for 1 hour and then incubated overnight with rabbit β-Actin (1:10000, Cat# AC206, ABclonal) and rabbit GSDME (1:1000, Cat# ab215191, Abcam) at 4 °C. After washing three times in TBS-T (TBS containing 0.05% Tween 20), the membrane was incubated with HRP goat anti-rabbit IgG H&L (1:2000, Cat# ab97051, Abcam) at room temperature for 2 h, and the signals were detected with an eECL Western Blot Kit (Cat# CW0049S, CWBIO, China) using a Tanon 5200 Multi Chemiluminescent Imaging System (Shanghai, China).

### Isolation of tfRFP^+^ microparticles

tfRFP-B16 cells were incubated with 10 μM melittin and 50 μg anti-tfRFP IgG in a 24-well plate for 4 hours. All samples were collected, cells were removed by low-speed centrifugation (2000 g, 1 min), the supernatant was centrifuged at high speed (9000 g, 7 mins), and the pellet was collected to obtain tfRFP^+^ microparticles.

### Flow cytometry

Mice were anesthetized with a mixture of 10 mg/kg xylazine and 100 mg/kg ketamine hydrochloride at the corresponding time points. After the mice were fully anesthetized, they were sacrificed by exsanguination. The liver and spleen of the mice were removed in a clean environment. These tissues were placed separately in DMEM low glucose medium (HyClone). After cutting the tissue with forceps and scissors, the livers were passed through 200-gauge stainless steel mesh and centrifuged at 100 g for 1 min. The supernatant was centrifuged at 1300 g for 5 mins. The resulting cell pellet was resuspended in 5 mL of 40% Percoll containing 0.5 mM EDTA, and centrifuged at 1260 g for 20 mins. The bottom layer cells were obtained and centrifuged with PBS containing 2% FBS. The blood cells were lysed in ACK buffer (155 mM NH_4_Cl, 10 mM KHCO_3_, 1 mM EDTA, 170 mM Tris, pH 7.3). The cells were washed once with PBS, and then Fixable Viability Dye eFluor 506 (Cat# 65-0866-18, Thermo Fisher, USA) was added and incubated for 30 mins. iNKT cells were labeled using PBS57-loaded APC-conjugated CD1d tetramer (NIH tetramer facility, USA). For other cell staining, the cells were incubated with anti-mouse CD16/32 (Clone 93, Cat# 101320, BioLegend, San Diego, CA, USA) for 10 mins at 4 °C, and then stained with the following anti-mouse antibodies: NK1.1-PE-Cy7 (Clone PK136, Cat# 108713, BioLegend), NK1.1-PE (Clone PK136, Cat# 108707, BioLegend), CD8a-Percp-Cy5.5 (Clone 53-6.7, Cat# 551162, BD Bioscience, San Jose, CA, USA), CD4-PE-Cy7 (Clone GK1.5 Cat# 100422, BioLegend), CD3-APC-Cy7 (Clone 145-2C11 Cat# 100329, BioLegend), and CD45-FITC (Clone 30-F11 Cat# 103107, BioLegend). For the detection of IFN-γ, Cell Activation Cocktail (with Brefeldin A) (Cat# 423303, BioLegend) was used, and the cells were fixed and then labeled with IFN-γ-PE (Clone XMG1.2 Cat# 505808, BioLegend). For the detection of Ki67, the cells were fixed and then labeled with Ki67-Brilliant Violet 421 (Clone 16A8 Cat# 652411, BioLegend). All the cells were obtained using a CytoFLEX flow cytometer (Beckman Coulter, Brea, CA, USA). The data were analyzed using FlowJo software (FlowJo, LLC, Ashland, USA).

### Immunofluorescence

For tissue samples, fresh mouse tissue was soaked in 4% paraformaldehyde (PFA) for 10 hours, and then sectioned at a thickness of 100 μm by a vibrating blade microtome (Leica VT1200, Germany). For cell samples, tumor cells were seeded on 24-well plates with a 14 mm diameter TC-treated cell imaging slides (Solarbio, Beijing, China) for adherent culture. Cells were treated with the corresponding drugs (50 μL tfRFP-immunized mouse serum, 50 μL normal mouse serum, 50 μg purified anti-tfRFP IgG, 500 μM SSA, 500 μM NAC and/or 15 μM Raptinal). Then, these slides were fixed in 4% PFA for 10 mins. The samples were blocked in PBS containing 0.02% Triton X-100 (Sigma, Missouri, USA) and 1% BSA for 1 h and then incubated with the primary antibody overnight at 4 °C. The anti-mouse antibodies used were as follows: NK1.1-Alexa Fluor 647 (Clone PK136, Cat# 108720, BioLegend), CD8a-Alexa Fluor 700 (Clone 53-6.7, Cat# 100724, BioLegend), Ly6G-Alexa Fluor 647 (Clone 1A8 Cat# 127610, BioLegend), F4/80-Alexa Fluor 647 (Clone BM8 Cat# 123122, BioLegend), B220-FITC (Clone RA3-6B2, Cat# 103205, BioLegend), B220-Alexa Fluor 647 (Clone RA3-6B2, Cat# 103226, BioLegend), anti-mouse IgG-DyLight 649 (Clone poly4053 Cat# 405312, BioLegend) and C5b-9-Alexa Fluor 647 (Clone 2A1 Cat# Sc-66190 AF647, Santa Cruz, Texas, USA). After washing off the unbound antibody and staining with DAPI, the samples were enclosed using PBS containing 50% glycerol (w/w). Samples were imaged under an LSM710 confocal microscope (Carl Zeiss, Germany) with a dry 20×/0.8 NA objective (Carl Zeiss), AX/AX R confocal microscope with a dry 40×/0.95 NA objective (Nikon, Japan) or spinning disk inverted confocal microscope (PerkinElmer, USA) with a dry 20×/0.75 NA objective (Olympus, Japan) or an oil 60x/1.42 NA objective (Olympus).

### Atomic force microscope (AFM) scanning the cell membrane

The surface morphology of the cell membrane was scanned by AFM as described in reference 60. Briefly, the tfRFP-B16 cells in the 24-well plate were treated with 500 μM SSA, or complete medium; the tfRFP-B16 cells, GSDME^-/-^tfRFP-B16 cells, and GSDME-GFP^mid^tfRFP-B16 cells were treated with 15 μM Raptinal; and the tfRFP-B16 cells were treated with 50 μg purified anti-tfRFP IgG, alone or in combination with 50 μL normal mouse serum, 50 μL heat-inactivated mouse serum, or 50 μL normal mouse serum plus 30 nM mouse Serpin G1. Then the treated cells on a 14 mm diameter slide were fixed with 4% PFA for 10 mins, washed with ultrapure water, and air-dried. The prepared cell samples were scanned using ScanAsyst intelligent imaging mode of Multimode 8 (Bruker, Germany). The AFM probe used in the present study was ScanAsyst-Air (Bruker, Germany). The scan parameters were as follows: the scan rate was set to 3 Hz, the ScanAsyst noise threshold was set to 5 nm, the peak force amplitude was set to 150 nm and the peak force frequency was set to 2 kHz. In this study, AFM was used to obtain the morphology of each group's cell membrane, and NanoScope Analysis 1.9 software (Bruker, Germany) was used to analyze and process the holes formed on each group's cell membrane.

### Live Cell Confocal Imaging

The 1 × 10^4^ tfRFP-B16 cells were cultured overnight in a chambered coverslip with 8 wells (ibidi, Germany). Then, 5 × 10^5^ bone marrow cells from ROSA^mT/mG^ mice were added with 50 μg anti-tfRFP IgG alone, or 50 μg anti-tfRFP IgG in combination with 50 μL normal mouse serum. After 4 hours, the cultured cells were imaged with a spinning disk inverted confocal microscope (PerkinElmer) with a dry 20×/0.75 NA objective (Olympus).

### Long-term intravital imaging of livers

Before performing long-term intravital imaging of the liver, mice needed to be assembled with a drawer-type abdominal window (DAW) 31. The mice that successfully assembled DAW were immobilized in custom-made boxes, exposing the imaging window. Then, intravital imaging was performed by a spinning disk inverted confocal microscope (PerkinElmer) with a dry 20×/0.75 NA objective (Olympus). Intravital imaging of liver metastases was performed on days 2/3/4/5/7 after tumor inoculation. During imaging, mice were maintained under anesthesia with 1.0% isoflurane in oxygen flow at 0.6 L/min controlled by a small animal anesthesia machine (RWD, Shenzhen, China) and maintained at a constant temperature of 37 °C.

### Liver intravital molecular imaging

The mice were anesthetized with a mixture of 10 mg/kg xylazine and 100 mg/kg ketamine hydrochloride. Next the abdominal cavity was depilated, and then the liver was exposed and pasted on a cover glass. The liver was attached to a coverslip, and then the skin outside the spleen was cut to expose the spleen. The mice were kept anesthetized by inhalation with 0.5% isoflurane in oxygen flow at 0.6 L/min through a small animal anesthesia machine (RWD), and the temperature was maintained at 37 °C with a thermostat. A spinning disk inverted confocal microscope (PerkinElmer) with a dry 20×/0.75 NA objective (Olympus) was used for imaging. Usually, the exposed left liver lobe of the mouse with relatively small jitter was selected for imaging and then scanned in the Z-axis direction at 3 μm intervals with 30 s or 60 s intervals. During the scanning process, about 2.5 × 10^6^ tumor cells were injected *via* the spleen, and a cotton swab was used to stop the bleeding. After the tumor was observed in the imaging field of view, continuous imaging was performed for at least 1 hour. The fluorescent signals of tfRFP were imaged using excitation at 561 nm, and the emissions were recorded at 680-730 nm. The fluorescent signals of CFP and FRET of C3 were imaged using excitation at 440 nm, and the emissions were recorded at 450-500 nm (CFP) and 510-560 nm (FRET). The fluorescent signals of F405 and F488 of HyPer7 were imaged using excitation at 405 nm (F405) and 488 nm (F488), and the emissions were recorded at 510-560 nm.

### Image data analysis

Image data were exported using Velocity (PerkinElmer, USA), and processed with FIJI (NIH, USA) and MATLAB (MathWorks, USA). We used the Calculator Plus tool in FIJI to calculate the ratio of the channel FRET to CFP or F488 to F405, and generate a new channel, named the “C3 Ratio” or “HyPer7 Ratio”. The formulae are as follows:



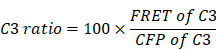





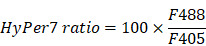



Then, we drew the outer contour of the tumor cell in each frame in FIJI. The selected cell needs to appear throughout the video at each frame, with a clear cell shape and a stable focal plane. Next, the segmented cells were added to ROI Manager in FIJI, and the mean gray value of the “C3 Ratio” or “HyPer7 Ratio” channel corresponding to each frame was obtained for each single cell. Finally, the graph was made in GraphPad Prism 8 (GraphPad Software, Inc., La Jolla, CA, USA) after batch processing in MATLAB.

### Statistical analysis

Experimental data are presented as means ± SEM. For comparisons of two groups, unpaired Student's* t*-test or the Mann-Whitney test was used. For comparisons of three or more groups, one-way ANOVA test followed by Tukey's multiple comparisons post-test, Brown-Forsythe and Welch ANOVA tests or the Kruskal-Wallis test followed by Dunn's multiple comparisons post-test was used. All statistical analyses were performed using GraphPad Prism 8. The statistical analysis is described in each figure legend. Differences between or among groups are denoted as ns for not significant, * for *P* < 0.05, ** for *P* < 0.01, *** for *P* < 0.001, and **** for *P* < 0.0001.

## Supplementary Material

Supplementary figures and movie legends.

Supplementary movie 1.

Supplementary movie 2.

Supplementary movie 3.

Supplementary movie 4.

Supplementary movie 5.

Supplementary movie 6.

Supplementary movie 7.

## Abbreviations

TAs: tumor antigens
intra-Ags: intracellular antigens
ROS: reactive oxygen species
MACs: membrane attack complexes
mAbs: monoclonal antibodies
ADCC: antibody-dependent cell-mediated cytotoxicity
ADCP: antibody-dependent cell phagocytosis
CDC: complement-dependent cytotoxicity
FDA: Food and Drug Administration
GSDME: Gasdermin E
tfRFP: tetrameric far-red fluorescent protein
CTLs: cytotoxic T lymphocytes
FRET: fluorescence resonance energy transfer
Ag: antigen
KCs: Kupffer cells
NAC: N-Acetyl-L-cysteine
SSA: sulfasalazine
GFP: green fluorescent protein
YFP: yellow fluorescent protein
CFP: cyan fluorescent protein
iNKT: invariant nature killer T
fDCs: follicular dendritic cells
ELISA: enzyme-linked immunosorbent assay
AFM: atomic force microscopy
BM: bone marrow
LSECs: liver sinusoidal endothelial cells
HE: hematoxylin and eosin
DAW: drawer-type abdominal window
tfRFP-IM: tfRFP-immunized mice
Non-IM: non-immunized mice
WT: wild-type
IFN-γ: interferon-γ
FBS: fetal bovine serum
PBS: phosphate buffered saline
PVDF: polyvinylidene fluoride
SDS-PAGE: sodium dodecyl sulfate polyacrylamide gel electrophoresis
SPF: specific pathogen-free
TBS: tris-buffered saline
WB: western blot
BSA: bovine serum albumin
IFA: incomplete freund's adjuvant
NA: numerical aperture

## References

[1] Saxena M, van der Burg SH, Melief CJM, Bhardwaj N (2021). Therapeutic cancer vaccines. Nat Rev Cancer.

[2] Coulie PG, Van den Eynde BJ, van der Bruggen P, Boon T (2014). Tumour antigens recognized by T lymphocytes: at the core of cancer immunotherapy. Nat Rev Cancer.

[3] Weiner LM, Murray JC, Shuptrine CW (2012). Antibody-based immunotherapy of cancer. Cell.

[4] Scott AM, Wolchok JD, Old LJ (2012). Antibody therapy of cancer. Nat Rev Cancer.

[5] Enokida T, Moreira A, Bhardwaj N (2021). Vaccines for immunoprevention of cancer. J Clin Invest.

[6] Matlung HL, Babes L, Zhao XW, van Houdt M, Treffers LW, van Rees DJ (2018). Neutrophils kill antibody-opsonized cancer cells by trogoptosis. Cell Rep.

[7] Guo K, Li J, Tang JP, Tan CPB, Hong CW, Al-Aidaroos AQO (2011). Targeting intracellular oncoproteins with antibody therapy or vaccination. Sci Transl Med.

[8] Gul N, Babes L, Siegmund K, Korthouwer R, Bogels M, Braster R (2014). Macrophages eliminate circulating tumor cells after monoclonal antibody therapy. J Clin Invest.

[9] Lofano G, Gorman MJ, Yousif AS, Yu WH, Fox JM, Dugast AS (2018). Antigen-specific antibody Fc glycosylation enhances humoral immunity via the recruitment of complement. Sci Immunol.

[10] Feng MY, Jiang W, Kim BYS, Zhang CC, Fu YX, Weissman IL (2019). Phagocytosis checkpoints as new targets for cancer immunotherapy. Nat Rev Cancer.

[11] Cheever MA, Allison JP, Ferris AS, Finn OJ, Hastings BM, Hecht TT (2009). The prioritization of cancer antigens: a national cancer institute pilot project for the acceleration of translational research. Clin Cancer Res.

[12] Uhlen M, Fagerberg L, Hallstrom BM, Lindskog C, Oksvold P, Mardinoglu A (2015). Tissue-based map of the human proteome. Science.

[13] Mullard A (2021). FDA approves 100th monoclonal antibody product. Nat Rev Drug Discov.

[14] Yang X, Xie S, Yang X, Cueva JC, Hou X, Tang Z (2019). Opportunities and challenges for antibodies against Intracellular antigens. Theranostics.

[15] Trenevska I, Li D, Banham AH (2017). Therapeutic antibodies against intracellular tumor antigens. Front Immunol.

[16] Wang Y, Gao W, Shi X, Ding J, Liu W, He H (2017). Chemotherapy drugs induce pyroptosis through caspase-3 cleavage of a gasdermin. Nature.

[17] Liu YY, Fang YL, Chen XF, Wang ZF, Liang XY, Zhang TZ (2020). Gasdermin E-mediated target cell pyroptosis by CAR T cells triggers cytokine release syndrome. Sci Immunol.

[18] Afshar-Kharghan V (2017). The role of the complement system in cancer. J Clin Invest.

[19] Serna M, Giles JL, Morgan BP, Bubeck D (2016). Structural basis of complement membrane attack complex formation. Nat Commun.

[20] Dudkina NV, Spicer BA, Reboul CF, Conroy PJ, Lukoyanova N, Elmlund H (2016). Structure of the poly-C9 component of the complement membrane attack complex. Nat Commun.

[21] Ritter AT, Shtengel G, Xu CS, Weigel A, Hoffman DP, Freeman M (2022). ESCRT-mediated membrane repair protects tumor-derived cells against T cell attack. Science.

[22] Zhang Y, Chen X, Gueydan C, Han J (2018). Plasma membrane changes during programmed cell deaths. Cell Res.

[23] Ammendolia DA, Bement WM, Brumell JH (2021). Plasma membrane integrity: implications for health and disease. BMC Biology.

[24] Piskounova E, Agathocleous M, Murphy MM, Hu Z, Huddlestun SE, Zhao Z (2015). Oxidative stress inhibits distant metastasis by human melanoma cells. Nature.

[25] Buchheit CL, Weigel KJ, Schafer ZT (2014). Cancer cell survival during detachment from the ECM: multiple barriers to tumour progression. Nat Rev Cancer.

[26] Weng XF, Bao ZZ, Wei XB (2021). Binary organic nanoparticles with enhanced reactive oxygen species generation capability for photodynamic therapy. J Innov Opt Heal Sci.

[27] Kubes P, Jenne C (2018). Immune responses in the liver. Annu Rev Immunol.

[28] Yang F, Liu S, Liu XL, Liu L, Luo MJ, Qi SH (2016). *In vivo* visualization of tumor antigen-containing microparticles generated in fluorescent-protein-elicited immunity. Theranostics.

[29] Qi SH, Li H, Lu LS, Qi ZY, Liu L, Chen L (2016). Long-term intravital imaging of the multicolor-coded tumor microenvironment during combination immunotherapy. eLife.

[30] Liu L, Dai BL, Li RX, Liu Z, Zhang ZH (2021). Intravital molecular imaging reveals the restrained capacity of CTLs in the killing of tumor cells in the liver. Theranostics.

[31] Deng DQ, Dai BL, Wei JS, Yuan XN, Yang XQ, Qi SH (2021). A drawer-type abdominal window with an acrylic/resin coverslip enables long-term intravital fluorescence/photoacoustic imaging of the liver. Nanophotonics.

[32] Heesters BA, Myers RC, Carroll MC (2014). Follicular dendritic cells: dynamic antigen libraries. Nat Rev Immunol.

[33] Hayes JD, Dinkova-Kostova AT, Tew KD (2020). Oxidative stress in cancer. Cancer Cell.

[34] Labuschagne CF, Cheung EC, Blagih J, Domart MC, Vousden KH (2019). Cell clustering promotes a metabolic switch that supports metastatic colonization. Cell Metab.

[35] Park HA, Brown SR, Kim Y (2020). Cellular mechanisms of circulating tumor cells during breast cancer metastasis. Int J Mol Sci.

[36] Trouw LA, Pickering MC, Blom AM (2017). The complement system as a potential therapeutic target in rheumatic disease. Nat Rev Rheumatol.

[37] Beinrohr L, Murray-Rust TA, Dyksterhuis L, Zavodszky P, Gal P, Pike RN (2011). Serpins and the complement system. Methods Enzymol.

[38] Chen ZY, Huang AF, Sun JY, Jiang TJ, Qin FX, Wu AP (2017). Inference of immune cell composition on the expression profiles of mouse tissue. Sci Rep.

[39] Chen ZY, Quan LJ, Huang AF, Zhao Q, Yuan Y, Yuan XY (2018). seq-ImmuCC: Cell-centric view of tissue transcriptome measuring cellular compositions of immune microenvironment from mouse RNA-seq data. Front Immunol.

[40] Ma S, Fu A, Chiew GG, Luo KQ (2017). Hemodynamic shear stress stimulates migration and extravasation of tumor cells by elevating cellular oxidative level. Cancer Lett.

[41] Zhou B, Zhang JY, Liu XS, Chen HZ, Ai YL, Cheng K (2018). Tom20 senses iron-activated ROS signaling to promote melanoma cell pyroptosis. Cell Res.

[42] Gao M, Yi J, Zhu J, Minikes AM, Monian P, Thompson CB (2019). Role of mitochondria in ferroptosis. Mol Cell.

[43] Yoon S, Kovalenko A, Bogdanov K, Wallach D (2017). MLKL, the Protein that mediates necroptosis, also regulates endosomal trafficking and extracellular vesicle generation. Immunity.

[44] Voskoboinik I, Whisstock JC, Trapani JA (2015). Perforin and granzymes: function, dysfunction and human pathology. Nat Rev Immunol.

[45] Luo HM, Yang J, Jin HL, Huang C, Fu JW, Yang F (2011). Tetrameric far-red fluorescent protein as a scaffold to assemble an octavalent peptide nanoprobe for enhanced tumor targeting and intracellular uptake *in vivo*. Faseb J.

[46] Ueno Y, Mio M, Sato C, Mio K (2007). Single particle conformations of human serum albumin by electron microscopy. J Electron Microsc.

[47] Ding J, Wang K, Liu W, She Y, Sun Q, Shi J (2016). Pore-forming activity and structural autoinhibition of the gasdermin family. Nature.

[48] Liu X, Xia S, Zhang Z, Wu H, Lieberman J (2021). Channelling inflammation: gasdermins in physiology and disease. Nat Rev Drug Discov.

[49] Headley MB, Bins A, Nip A, Roberts EW, Looney MR, Gerard A (2016). Visualization of immediate immune responses to pioneer metastatic cells in the lung. Nature.

[50] Cranmer SL, Ashworth KJ, Yao Y, Berndt MC, Ruggeri ZM, Andrews RK (2011). High shear-dependent loss of membrane integrity and defective platelet adhesion following disruption of the GPIbalpha-filamin interaction. Blood.

[51] Bogers WM, Stad RK, Van Es LA, Daha MR (1992). Both Kupffer cells and liver endothelial cells play an important role in the clearance of IgA and IgG immune complexes. Res Immunol.

[52] Georgiannakis A, Burgoyne T, Lueck K, Futter C, Greenwood J, Moss SE (2015). Retinal pigment epithelial cells mitigate the effects of complement attack by endocytosis of C5b-9. J Immunol.

[53] Xie CB, Jane-Wit D, Pober JS (2020). Complement membrane attack complex: new roles, mechanisms of action, and therapeutic targets. Am J Pathol.

[54] Miyabe Y, Miyabe C, Murooka TT, Kim EY, Newton GA, Kim ND (2017). Complement C5a receptor is the key initiator of neutrophil adhesion igniting immune complex-induced arthritis. Sci Immunol.

[55] Huppert LA, Green MD, Kim L, Chow C, Leyfman Y, Daud AI (2022). Tissue-specific Tregs in cancer metastasis: opportunities for precision immunotherapy. Cell Mol Immunol.

[56] Crispe IN (2014). Immune tolerance in liver disease. Hepatology.

[57] Pak VV, Ezerina D, Lyublinskaya OG, Pedre B, Tyurin-Kuzmin PA, Mishina NM (2020). Ultrasensitive genetically encoded indicator for hydrogen peroxide identifies roles for the oxidant in cell migration and mitochondrial function. Cell Metab.

[58] Susaki EA, Tainaka K, Perrin D, Kishino F, Tawara T, Watanabe TM (2014). Whole-brain imaging with single-cell resolution using chemical cocktails and computational analysis. Cell.

[59] Yu X, Chen L, Liu JQ, Dai BL, Xu GQ, Shen GX (2019). Immune modulation of liver sinusoidal endothelial cells by melittin nanoparticles suppresses liver metastasis. Nat Commun.

[60] Luo HM, Yang J, Jin HL, Huang C, Fu JW, Yang F (2019). Visualization of perforin/gasdermin/complement-formed pores in real cell membranes using atomic force microscopy. Cell Mol Immunol.

