# Supplementary material for: Intravital molecular imaging reveals that ROS-caspase-3-GSDME-induced cell punching enhances humoral immunotherapy targeting intracellular tumor antigens
Source: Theranostics. 2022 Oct 31;12(17):7603–23. doi: 10.7150/thno.75966 (PMC9691348; doi:10.7150/thno.75966)
Supplement: Supplementary file 1 — Supplementary figures and movie legends. [file thnov12p7603s1.pdf]

# Intravital molecular imaging reveals that ROS-caspase-3-GSDME-induced cell punching enhances humoral immunotherapy targeting intracellular tumor antigens

Bolei Dai,[1]† Ren Zhang,[1]† Shuhong Qi,[1]* Lei Liu,[1] Xian Zhang,[1] Deqiang Deng,[1] Jie Zhang,[1] Yilun Xu,[2] Fanxuan Liu,[1] Zheng Liu,[1] Qingming Luo,[2] and Zhihong Zhang[1,2]*

[1]Britton Chance Center and MoE Key Laboratory for Biomedical Photonics, Wuhan National Laboratory for Optoelectronics-Huazhong University of Science and Technology, Wuhan, Hubei 430074, China

[2]School of Biomedical Engineering, Hainan University, Haikou, Hainan 570228, China

† These authors contributed equally to this work

* Correspondence: Zhihong Zhang, czyzzh@mail.hust.edu.cn;
Shuhong Qi, qishuhong@hust.edu.cn;

Address: Room G304, Britton Chance Center for Biomedical Photonics, Wuhan National Laboratory for Optoelectronics-Huazhong University of Science and Technology, Wuhan, Hubei 430074, China

Fax: +86-27-87792034; Tel: +86-27-87792033;

Zhihong Zhang (czyzzh@mail.hust.edu.cn) and Shuhong Qi (qishuhong@hust.edu.cn) are the corresponding authors for communication with the Editorial and Production offices.

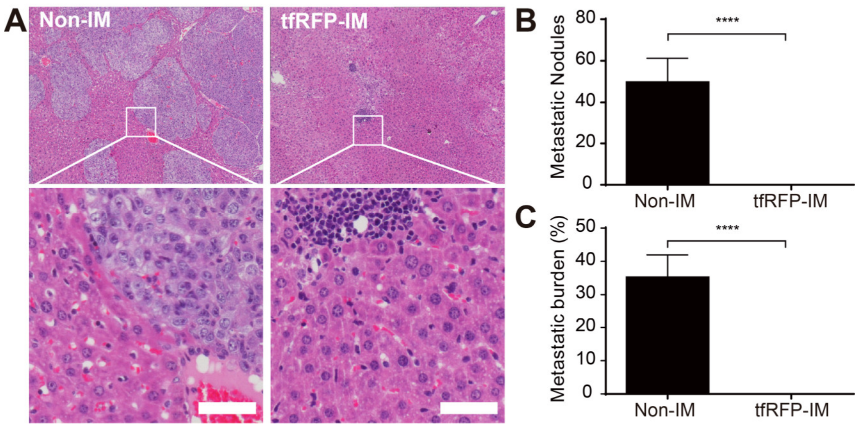


**Figure S1. Hematoxylin and Eosin (HE) staining of livers from tfRFP-immunized and non-immunized mice.** (**A**) A representative image of liver HE staining in tfRFP-immunized and non-immunized groups. Scale bar in the large image, 300 µm. Scale bar in the enlarged image, 50 µm. (**B** and **C**) Quantification of metastatic nodules and metastatic burden in (**A**). Data from 5 livers; each had five lobes. Data are presented as mean ± SEM. Statistical analysis was performed using the Mann Whitney test, ****$P < 0.0001$.

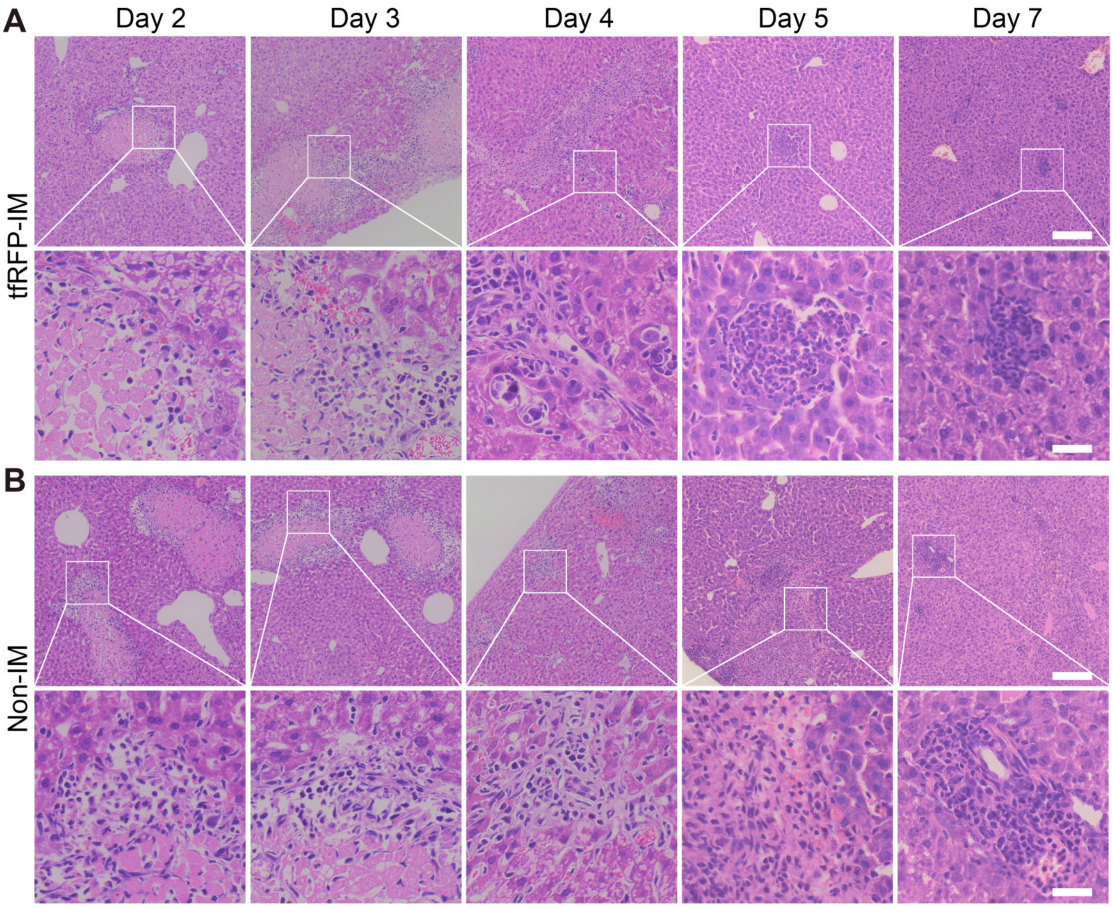


**Figure S2. Representative HE staining sections of livers on Day 2/3/4/5/7 after tfRFP-B16 tumor cells injection.** The images are from (**A**) tfRFP-immunized mice and (**B**) non-immunized mice. The up rows are large images, Scale bar, 150 μm. The bottom rows are enlarged images, Scale bar, 30 μm.

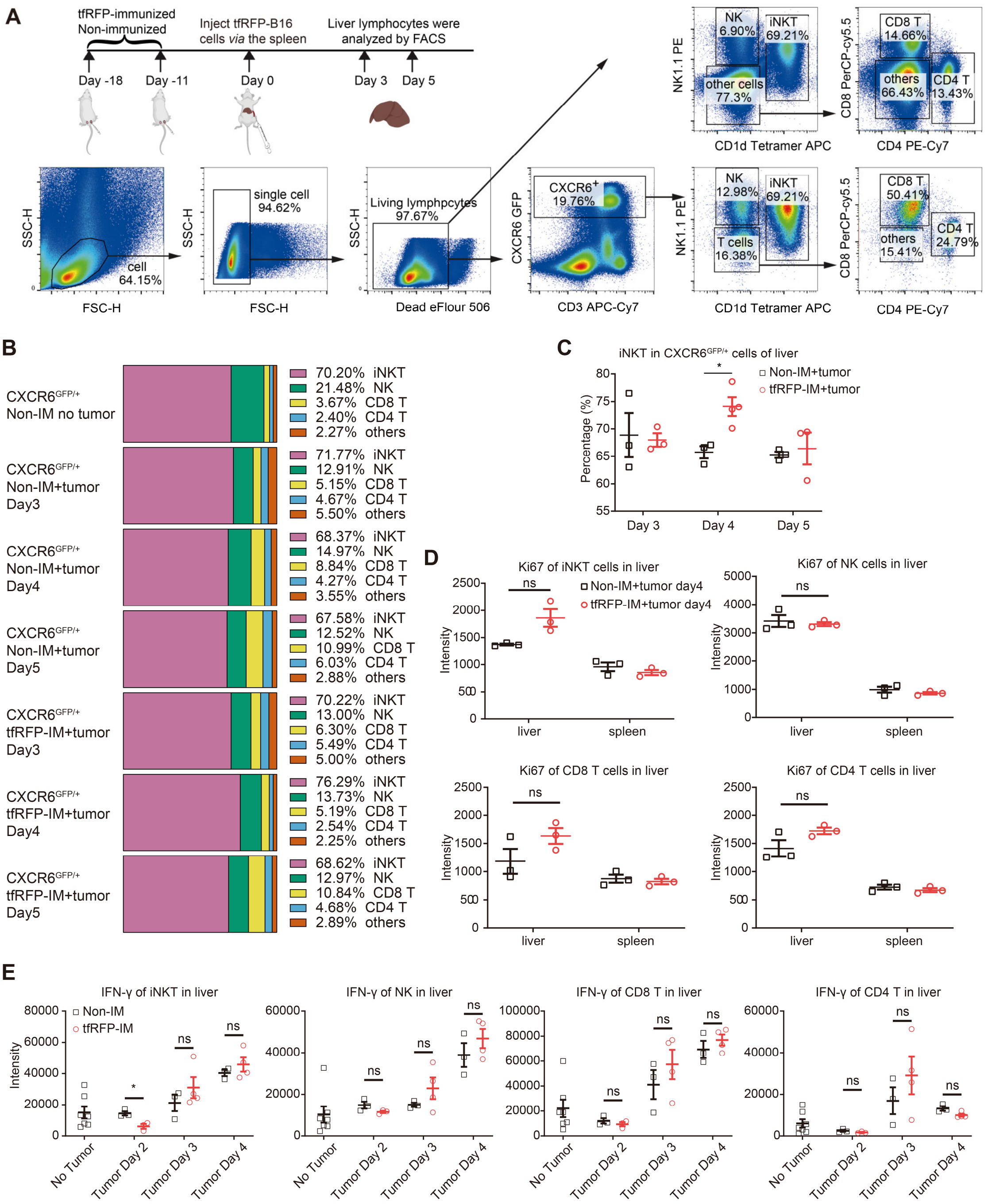


**Figure S3. Flow cytometry analysis of hepatic lymphocytes of livers at different days after tumor cells incubation.** (**A**) Schedules for liver lymphocytes FACS analysis after liver metastases and flow cytometry logic gates. (**B**) The composition ratio of lymphocytes in the hepatic CXCR6-GFP cells of tfRFP-immunized and non-immunized mice after tfRFP-B16 cell inoculation on different days (n = 3 per group). CXCR6$^{GFP/+}$ Non-IM mice without tumor served as normal control group. (**C**) The proportion of iNKT cells in CXCR6-GFP cells on different inoculation days in tfRFP-immunized and non-immunized mice. Each data point represents data from a mouse. Data are presented as mean ± SEM. Statistical analysis was performed using the unpaired Student's *t*-

test, *$P$ < 0.05. (**D**) Ki67 expression of lymphocytes in the liver of tfRFP-immunized and non-immunized mice on day 4 after tfRFP-B16 injection. Each data point represents data from a mouse. Data are presented as mean ± SEM. Statistical analysis was performed using the Mann Whitney test, ns: not significant. (**E**) IFN-γ expression of lymphocytes in the liver of tfRFP-immunized and non-immunized mice on day 4 after tfRFP-B16 injection. Data are presented as mean ± SEM. Statistical analysis was performed using the Mann Whitney test, ns: not significant, *$P$ < 0.05.

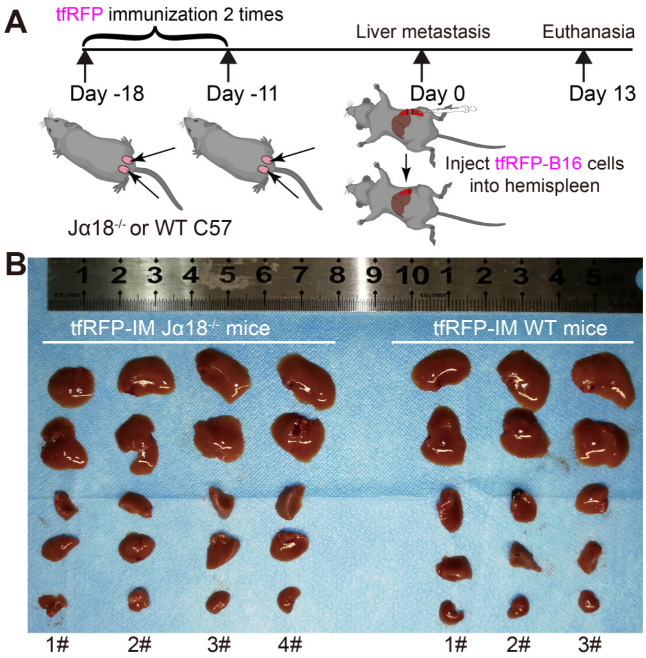


**Figure S4. iNKT cell deficiency did not affect the clearing of tfRFP-B16 by the tfRFP-elicited immune response.** (**A**) Schedules for the liver metastasis model in iNKT cell-deficient mice (Jα18$^{-/-}$) or wild-type mice (WT) after tfRFP-immunization. (**B**) Representative white-light imaging of the 5 lobes of livers from iNKT cell-deficient mice (Jα18$^{-/-}$) or wild-type mice (WT) after tfRFP-immunization.

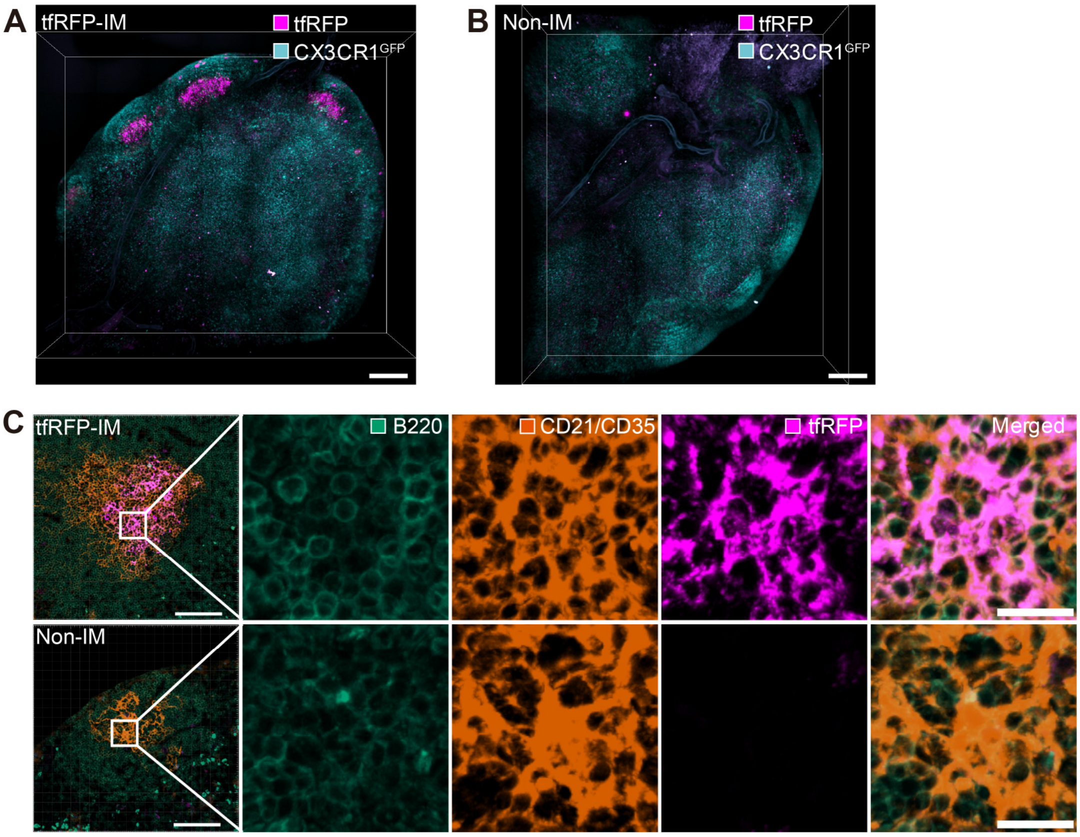


**Figure S5. tfRFP is stored in fDCs in the germinal centers of inguinal lymph nodes.** (**A and B**) Representative 3D images of inguinal lymph nodes from mice 18 days after the first immunization; (**A**) is the tfRFP-immunized group, and (**B**) is the non-immunized group. Scale bar, 300 μm. (**C**) Representative immunofluorescence images of inguinal lymph nodes in tfRFP-immunized mice (top row) and the non-immunized mice (bottom row). Scale bar of large field images, 50 μm. Scale bar of enlarged images, 10 μm.

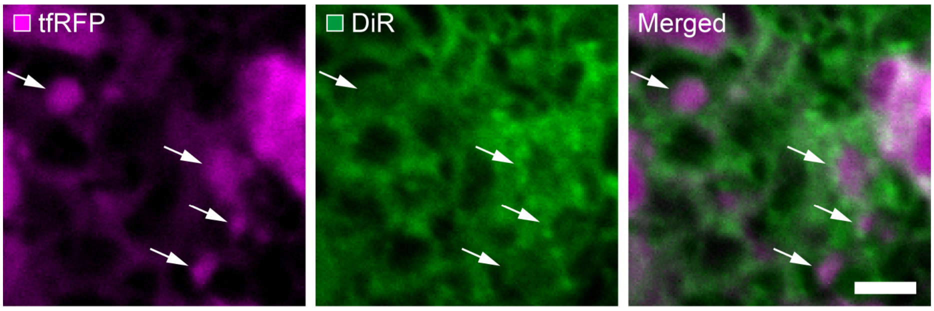


**Figure S6. tfRFP$^+$ particles in the liver of non-immunized mice have a membrane structure.** Representative tissue fluorescence confocal images of the tfRFP-B16 cells forming tfRFP$^+$ particles, which were stained with DiR (a membrane dye). The white arrow indicates DiR positive tfRFP$^+$ particles in the non-immunized group. Scale bar, 10 μm.

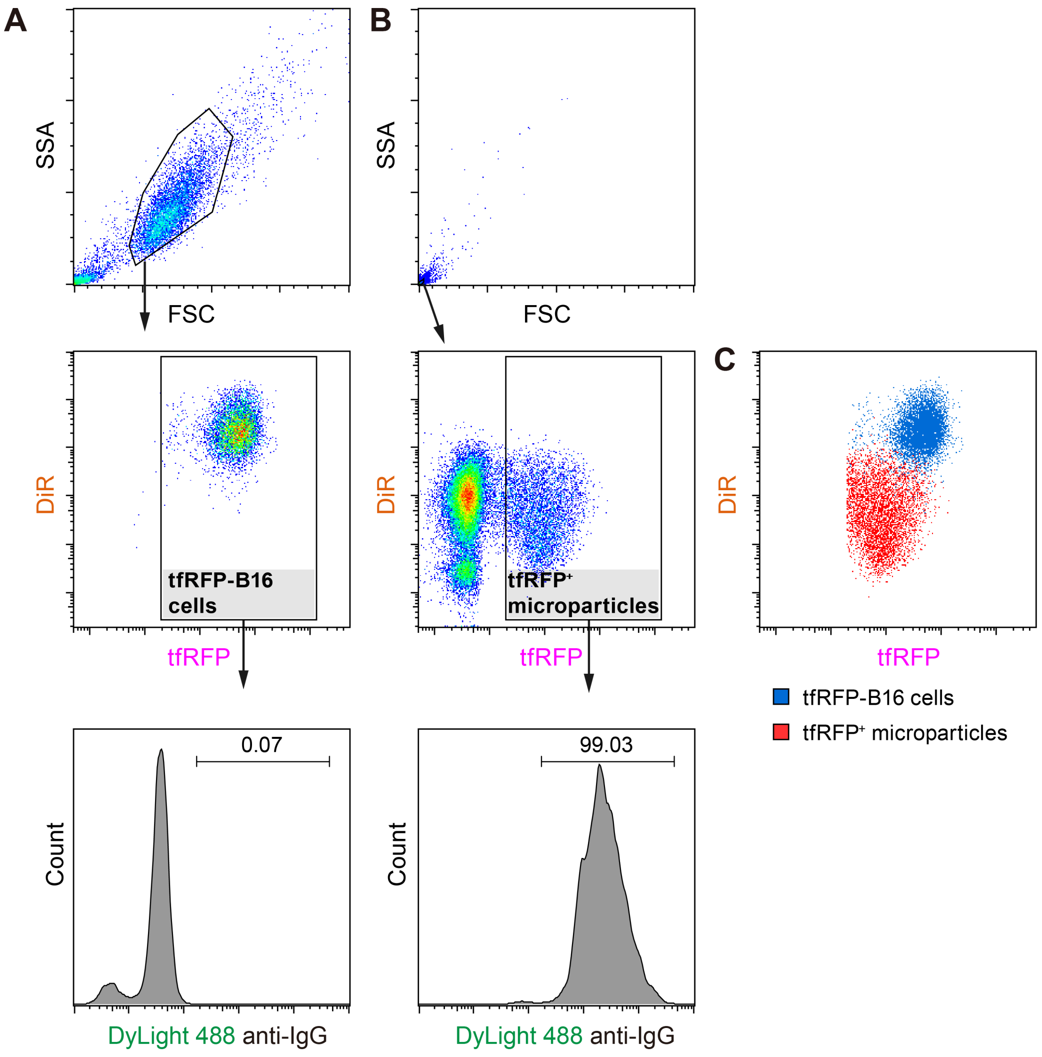


**Figure S7. Flow cytometry verified tfRFP$^+$ microparticles were antigen-antibody complexes.** **(A)** Flow cytometry results of untreated tfRFP-B16 cells labeled with Dylight 488 anti-IgG and the membrane dye DiR. (**B**) Flow cytometry results of tfRFP$^+$ microparticles (from supernatant of tfRFP-B16 cells treated with melittin and anti-tfRFP IgG) labeled with DyLight 488 anti-IgG and the membrane dye DiR. (**C**) Flow scatter plot of a mixture of tfRFP$^+$ microparticles and tfRFP-B16 cells. tfRFP-B16 cells were in blue and tfRFP$^+$ microparticles were in red.

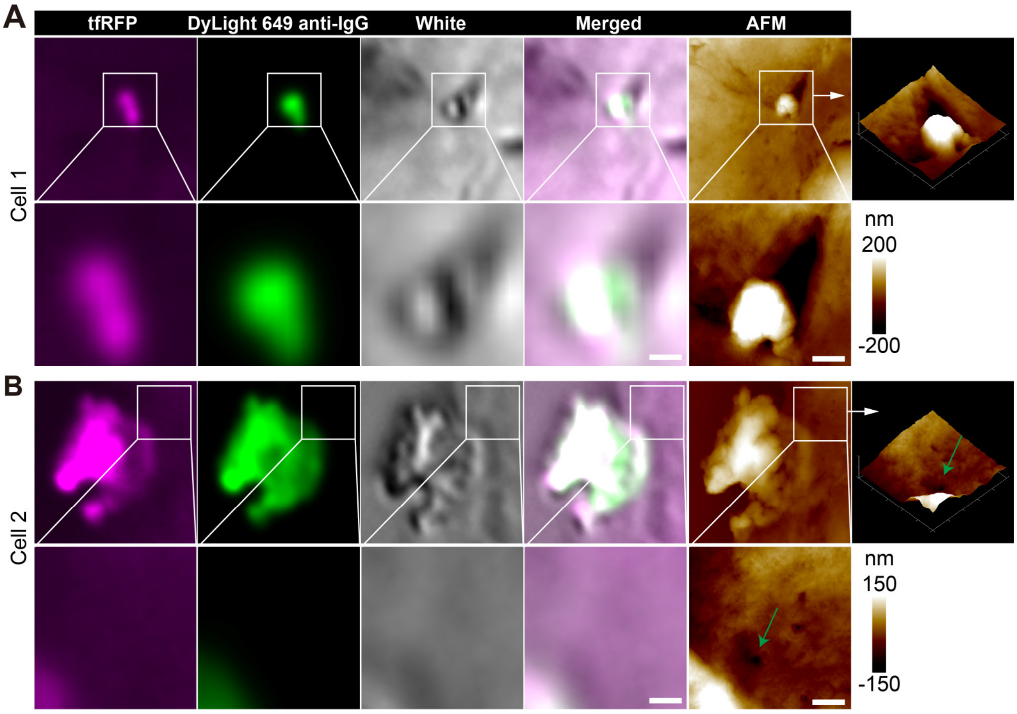


**Figure S8. Representative confocal images and AFM images of the same cells stained with the DyLight 649 anti-mouse IgG antibody.** (**A**) Representative immunofluorescence confocal imaging and AFM imaging of two tfRFP-B16 cells stained with the DyLight 649 anti-mouse IgG antibody after treatment with tfRFP-immunized serum for 4 hours. Scale bar, 1 μm. (**B**) Representative immunofluorescence confocal imaging and AFM imaging of two tfRFP-B16 cells stained with the DyLight 649 anti-mouse IgG antibody after treatment with tfRFP-immunized serum for 4 hours. The green arrow in the AFM image points to the hole near the $tfRFP^{+}$ microparticle. Scale bar, 1 μm.

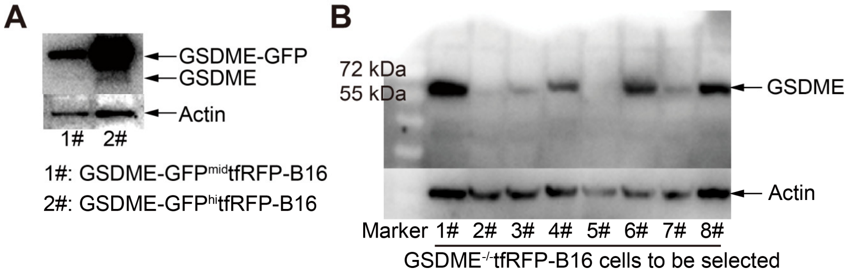


**Figure S9. Identification of GSDME expression in GSDME-GFP$^{mid}$tfRFP-B16, GSDME-GFP$^{hi}$tfRFP-B16 and GSDME$^{-/-}$tfRFP-B16 cells.** (**A**) Western blot analysis of GSDME-GFP$^{mid}$tfRFP-B16 cells and GSDME-GFP$^{hi}$tfRFP-B16 cells. (**B**) Western blot analysis of candidate GSDME$^{-/-}$tfRFP-B16 cells. β-Actin was used as the loading control in both cell groups.

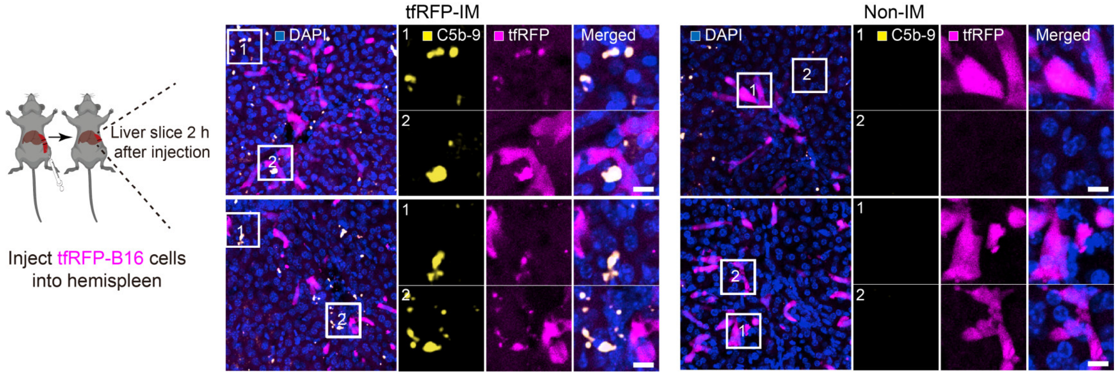


**Figure S10. Complement membrane attack complex formed on tfRFP-B16 cells in the tfRFP-immunized group.** Representative immunofluorescence images of C5b-9 in the liver 2 hours after tumor cell injection. Scale bar, 10 µm.

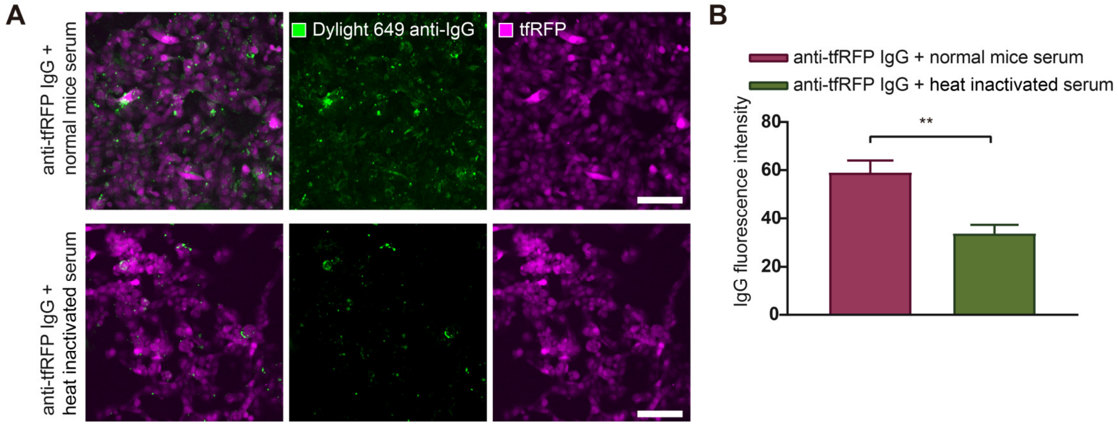


**Figure S11. The complement system enhanced anti-tfRFP IgG binding to the cell membranes.** (**A**) Representative confocal images of cultured tfRFP-B16 cells after 4 hours of incubation with anti-tfRFP IgG plus normal mouse serum or anti-tfRFP IgG plus heat-inactivated mouse serum. (**B**) Statistics of the fluorescence intensity of the IgG channel in (**A**). Data are presented as mean ± SEM. Statistical analysis was performed using unpaired Student's *t*-test, $^{**}P < 0.01$.

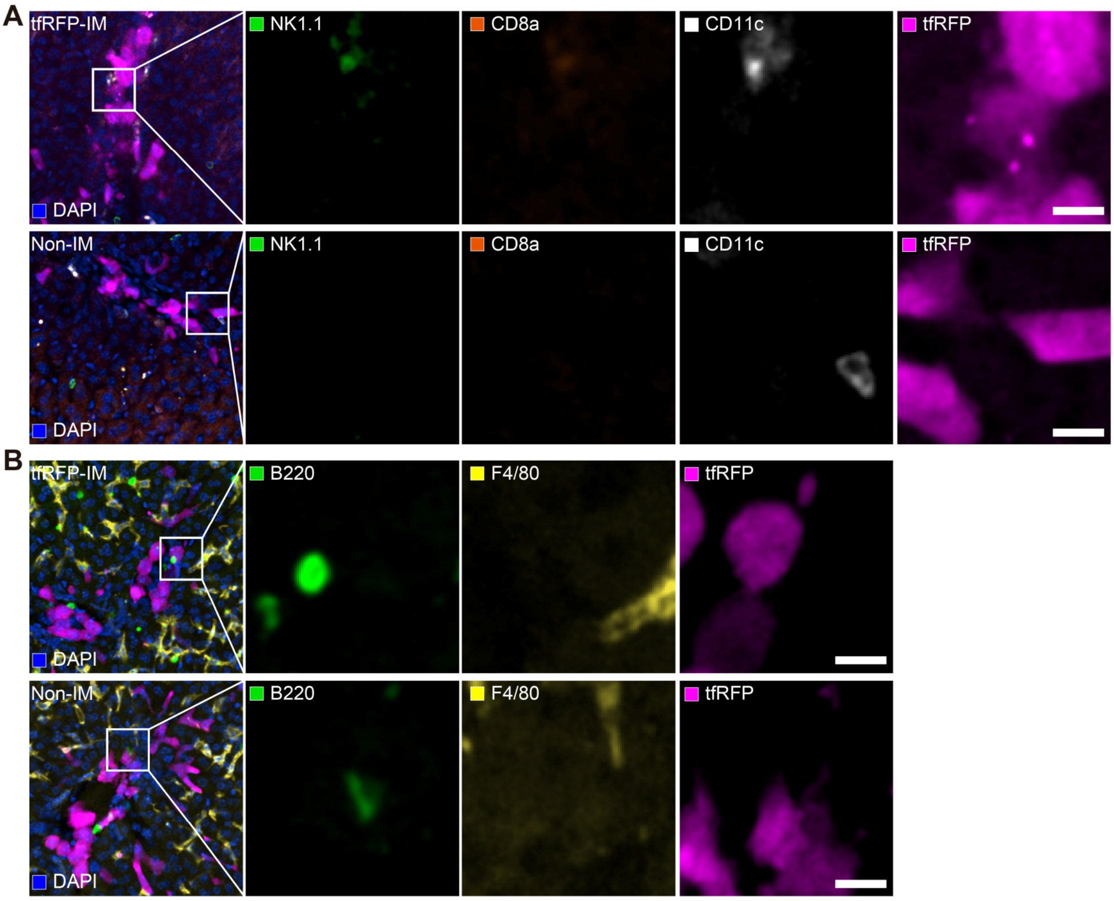


**Figure S12. NK cells, CD8$^+$ T cells, B cells, DCs, and macrophages were not recruited in the early stage.** (**A**) Representative images of NK cells (NK1.1$^+$), CD8$^+$ T cells (CD8a$^+$) and DCs (CD11c$^+$) near the tfRFP-B16 tumor cells 2 hours after tfRFP-B16 cells injection. Scale bar, 10 µm. (**B**) Representative images of B cells (B220$^+$), and macrophages (F4/80$^+$) near the tfRFP-B16 tumor cells 2 hours after tfRFP-B16 cells injection. Scale bar, 10 µm.

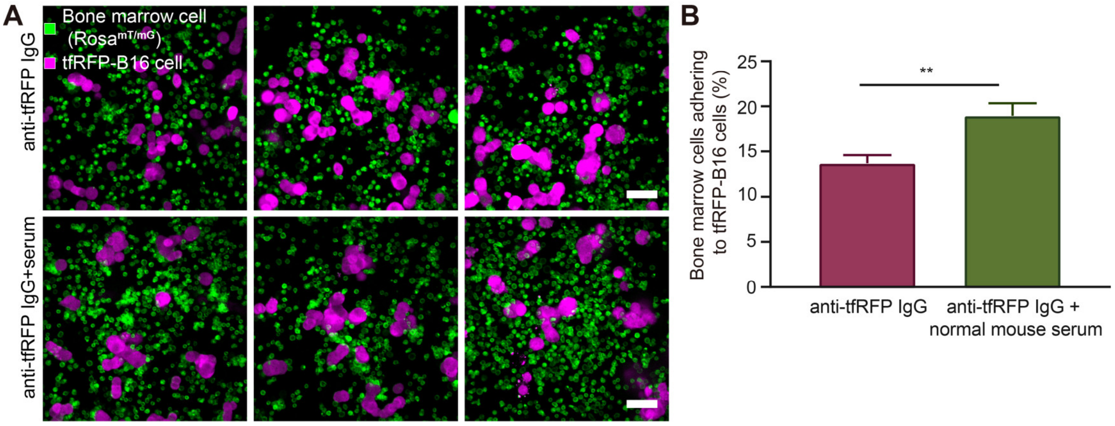


**Figure S13.** ***In vitro*** **cell experiment of neutrophils recruitment.** (A) Representative live cell confocal images of tfRFP-B16 cells co-cultured with bone marrow cells (about 60% of them were neutrophils) from ROSA$^{mT/mG}$ mice were treated with anti-tfRFP IgG plus normal mouse serum or anti-tfRFP IgG alone for 2 hours. Scale bar, 50 μm. (**B**) Statistics of the proportion of bone marrow cells contacted with tfRFP-B16 cells in all bone marrow cells. Data are presented as mean ± SEM. Statistical analysis was performed using the unpaired Student's $t$ test, $^*P < 0.05$.

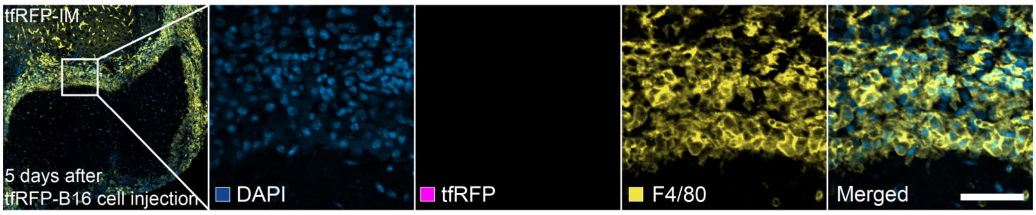


**Figure S14. Macrophages surround cleared liver metastasis.** F4/80$^{+}$ macrophages formed a circle at the site of tfRFP-B16 cell clearance in livers of tfRFP-immunized mice on day 5 after tfRFP-B16 cells injection. Scale bar, 100 µm.

**Movie S1: *In vivo* time-lapse imaging of the infiltrated CXCR6-GFP cells and tfRFP$^+$ microparticles in tfRFP-B16 metastasis of liver in tfRFP-immunized mice on Day 3.** Time-lapse intravital imaging was acquired by oil 60×/1.42 NA objective. CXCR6-GFP cells are shown in green, and tfRFP-B16 cells and tfRFP$^+$ microparticles are shown in magenta. Scale bar, 20 μm.

**Movie S2: *In vivo* time-lapse imaging of the disruption of tfRFP-B16 cells to form tfRFP$^+$ microparticles within the first hour of tfRFP-B16 inoculation in the tfRFP-immunized mice.** Time-lapse intravital imaging was acquired by air 20×/0.75 NA objective. tfRFP-B16 cells and tfRFP$^+$ microparticles are shown in magenta. Scale bar, 30 μm.

**Movie S3: *In vivo* time-lapse imaging of the formation of tfRFP$^+$ particles within the first hour of tfRFP-B16 inoculation in the non-immunized mice.** Time-lapse intravital imaging was acquired by air 20×/0.75 NA objective. tfRFP-B16 cells and tfRFP$^+$ particles are shown in magenta. Scale bar, 30 μm.

**Movie S4: *In vivo* time-lapse imaging of the disruption of tfRFP-B16 cells with tfRFP disappearance within the first hour of tfRFP-B16 inoculation in the non-immunized mice.** The time-lapse intravital imaging was acquired by air 20×/0.75 NA objective. tfRFP-B16 cells are shown in magenta. Scale bar, 30 μm.

**Movie S5: *In vivo* time-lapse imaging of the livers in non-immunized mice injected with a mixture of a 1:1 ratio of MLS-HyPer7-B16 cells and tfRFP-C3-B16 cells via the spleen.** The top row shows fluorescence images, the middle row shows the HyPer7 ratio (F488/F405) images of MLS-HyPer7-B16 cells, and the bottom row shows the C3 ratio (FRET/CFP) images of tfRFP-C3-B16 cells. Time-lapse intravital imaging was acquired by air 20×/0.75 NA objective. Rosa-mTom cells are shown in blue, tfRFP-C3-B16 tumor cells are shown in magenta, and MLS-HyPer7-B16 cells are shown in green. Scale bar, 20 μm.

**Movie S6: *In vivo* time-lapse imaging of the livers in non-immunized mice injected with a**

**mixture of a 1:1 ratio of MLS-HyPer7-B16 cells and tfRFP-C3-B16 cells via the spleen after injection of NAC.** The top row shows fluorescence images, the middle row shows the HyPer7 ratio (F488/F405) images of MLS-HyPer7-B16 cells, and the bottom row shows the C3 ratio (FRET/CFP) images of tfRFP-C3-B16 cells. Time-lapse intravital imaging was acquired by air 20×/0.75 NA objective. Rosa-mTom cells are shown in blue, tfRFP-C3-B16 tumor cells are shown in magenta, and MLS-HyPer7-B16 cells are shown in green. Scale bar, 20 μm.

**Movie S7: *In vivo* time-lapse imaging of the liver within one hour after injecting tfRFP-immunized mice with GSDME-GFP$^{hi}$tfRFP-B16 cells.** GSDME-GFP is shown in green, and tfRFP is shown in magenta. Scale bar, 20 μm.
